# A roadmap for medical large language models: a review of foundations, applications, and challenges

**DOI:** 10.1016/j.mmr.2026.100050

**Published:** 2026-06-27

**Authors:** Yu-Yang Sha, Li Yu, Ze-Hui Lin, Amandeep Kaur, Yan-Yan Lou, Shivanand S Gornale, Tian-Yu Zhang, Ling Shing Wong, Zhi-Wen Wang, Yan Yan, Xian-Bin Zhang, Rui Hong, Ka Li, Sio Kei Im, Paulo de Carvalho, Tao Tan, Ke-Feng Li

**Affiliations:** a Faculty of Applied Sciences, Macao Polytechnic University, Macau 999078, Macao SAR, China; b Department of Oncology, Shengjing Hospital of China Medical University, Shenyang 110004, China; c Department of Computer Science and Technology, School of Engineering and Technology, Central University of Punjab, Bathinda, Punjab 151401, India; d Division of Hematology and Oncology, Mayo Clinic, Jacksonville, FL 32224, USA; e Department of Computer Science, School of Mathematics and Computing Sciences, Rani Channamma University, Belagavi, Karnataka 590016, India; f Department of Radiology, the Netherlands Cancer Institute, Amsterdam 1066 CX, the Netherlands; g Faculty of Health and Life Sciences, INTI International University, Nilai, Negeri Sembilan 71800, Malaysia; h School of Nursing, Peking University, Beijing 100191, China; i Guangdong-Hong-Kong-Macao University Joint Laboratory of Interventional Medicine, the Fifth Affiliated Hospital, Sun Yat-Sen University, Zhuhai 519000, Guangdong, China; j Department of General Surgery and Institute of Precision Diagnosis and Treatment of Digestive System Tumors, Carson International Cancer Center, Shenzhen University General Hospital, Shenzhen University, Shenzhen 518055, Guangdong, China; k Medicine and Engineering Interdisciplinary Research Laboratory of Nursing and Materials, West China Hospital, Sichuan University, Chengdu 610041, China; l Institute of Biomedical Engineering, College of Medicine, Southwest Jiaotong University, Chengdu 610031, China; m Informatics Engineering Department, Faculty of Science and Technology, University of Coimbra, 3030-290 Coimbra, Portugal

**Keywords:** Medical large language models (Med-LLMs), Artificial intelligence (AI), Clinical applications, Model evaluation, Clinical deployment

## Abstract

Medical large language models (Med-LLMs) have shown considerable promise across a broad range of clinical tasks, including decision support, medical documentation, patient communication, multimodal analysis, and telemedicine. Their rapid development has generated growing interest in how large language models (LLMs) may support healthcare practice, while also raising important questions about reliability, clinical validity, and safe deployment. This review provides a structured overview of recent progress in Med-LLMs by examining their major application areas, key challenges, and emerging future directions. Current evidence shows that the clinical usefulness of Med-LLMs cannot be judged by model performance alone. Their value in practice depends on whether they are supported by reliable evidence, remain consistent with current medical knowledge, and can be integrated into clinical workflows. Important challenges remain in evaluation, safety, knowledge updating, and real-world deployment. These issues reflect a gap between performance in controlled settings and clinical practice. Future progress will require stronger clinical validation, better alignment with medical practice, and more careful deployment across different settings. The clinical impact of Med-LLMs will depend on whether they can be used as reliable tools in clinical care.

## 1 Background

The launch of ChatGPT by OpenAI on November 30, 2022, marked a pivotal milestone in artificial intelligence (AI), propelling large language models (LLMs) to global prominence [1]. Unlike earlier breakthroughs such as AlphaFold [2], which primarily garnered attention within specific scientific communities, ChatGPT demonstrated exceptional capabilities in natural language understanding (NLU) and generation (NLG), capturing widespread interest across academia, industry, and the general public [3]. LLMs now exhibit sophisticated abilities in contextual reasoning, coherent text generation, and multi-turn dialogue, eliciting intense discussions regarding the future direction and societal implications of artificial general intelligence (AGI) [4]. The introduction of LLMs has not only transformed human-computer interaction but also catalyzed a surge of interdisciplinary research aimed at enhancing model capabilities, ensuring safety, and aligning AI behavior with human ideals [5]. Consequently, LLMs are increasingly recognized as functional infrastructure for building more adaptive, generalizable, and cognitively intelligent systems.

**Fig. 1:**
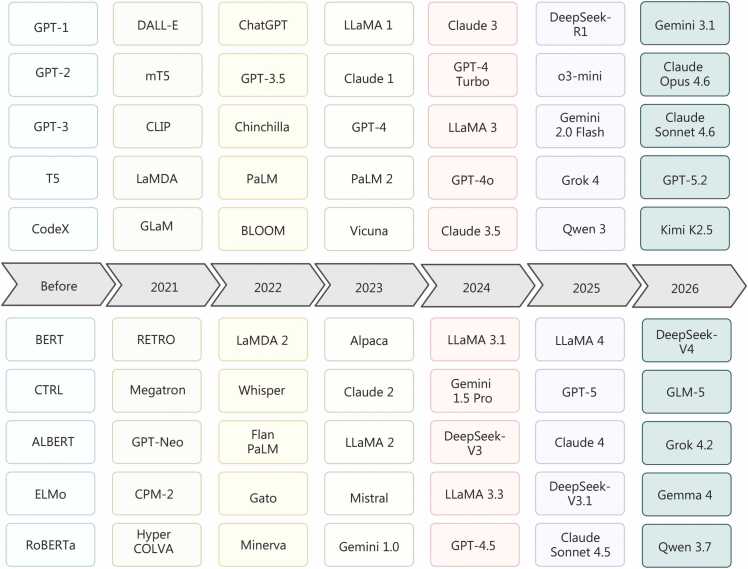
Chronological overview of major large language models (LLMs) developed in recent years. ALBERT. A lite BERT; BERT. Bidirectional encoder representations from transformers; CLIP. Contrastive language-image pre-training; CTRL. Conditional transformer language model; ELMo. Embeddings from language model; GPT. Generative pre-trained transformer; LLaMA. Large language model meta AI; PaLM. Pathways language model; T5. Text-to-text transfer transformer.

In recent years, research on LLMs has expanded rapidly, with leading research institutions and technology companies releasing models such as LLaMA, Mistral, Qwen, DeepSeek, Gemini, and Grok [6], [7], [8], [9]. These models demonstrate strong performance across diverse natural language processing (NLP) tasks such as generation, translation, summarization, and information extraction. Fig. 1 illustrates the chronological evolution of major LLMs, highlighting continuous architectural refinement and capability expansion. A central driver of this progress is the scaling law, which suggests that increasing the model size and training data often leads to substantial enhancements in model performance [10]. Under this paradigm, numerous organizations have pushed the boundaries of model size. For instance, the LLaMA family currently covers model sizes ranging from approximately 7B to 405B parameters [8], [11], [12], while DeepSeek-V3 has been reported as a mixture of experts (MoE) model with 671B total parameters [13]. Beyond parameter scaling alone, emerging paradigms such as MoE architectures and retrieval-augmented generation (RAG) frameworks are increasingly explored to improve efficiency, adaptability, and domain specialization, signaling important directions for the next generation of LLMs [14], [15].

Building upon the success of general-purpose LLMs, research has increasingly shifted toward domain-specific applications, including disease diagnosis, medical image understanding, and other clinically oriented tasks [16], [17], [18]. Prior studies suggest that LLM-based systems can achieve competitive or improved performance over traditional AI methods in certain medical tasks, benefiting from their contextual reasoning and language comprehension capabilities [3], [4], [16]. Among these domains, the medical domain has garnered particular attention due to its crucial societal importance and the intrinsic complexity of clinical reasoning. Clinical decision-making often involves uncertainty, multi-step inference, and risk-sensitive judgments that directly affect patient outcomes. Consequently, AI systems deployed in such settings must satisfy stringent requirements for safety, privacy, reliability, and accountability [5]. These challenges have motivated the development of medical LLMs (Med-LLMs), which are specifically adapted to medical knowledge and clinical workflows and are increasingly explored as intelligent support systems for clinical practice [19], [20].

Med-LLMs are typically derived from general-purpose foundation models and further adapted for medical use through domain-adaptive pretraining and fine-tuning strategies. These strategies include supervised fine-tuning (SFT), parameter-efficient fine-tuning (PEFT), and instruction tuning and domain alignment techniques [21], [22]. Such adaptations improve the models’ understanding of medical terminology, clinical narratives, and diagnostic reasoning patterns. In addition, RAG and domain-specific knowledge integration are increasingly incorporated to enhance factual grounding and reduce hallucinations in high-stakes clinical settings [15], [23]. Early applications of these adaptation strategies led to representative Med-LLMs such as ChatDoctor, MedAlpaca, and PMC-LLaMA, which demonstrated the feasibility of aligning general-purpose LLMs with medical knowledge and clinical dialogue tasks [21], [24], [25]. Building upon these initial efforts, recent advancements have moved Med-LLMs beyond early experimental prototypes toward more clinically oriented and robust systems. For example, the Me-LLaMA family builds on open-source LLaMA models with continued medical pretraining and demonstrates strong capability in medical text analysis tasks [26]. Hulu-Med represents a multimodal extension, integrating medical text with imaging and video to support more complex clinical reasoning [27]. Variants of general models, such as MedGemma derived from multimodal Gemma architectures, are also being explored for clinical assistance and health-oriented analysis [28]. Together, these developments indicate that Med-LLMs are evolving toward systems with practical relevance for medical practice and healthcare delivery. Nevertheless, ensuring their safe and reliable deployment in real-world medical environments remains a critical challenge, motivating the structured analysis presented in this review.

Despite rapid progress, translating Med-LLMs from promising research prototypes into routine medical practice remains non-trivial. Most current models are still trained primarily for next-token prediction, which supports fluent generation but may be brittle under the uncertainty, causal reasoning, and multi-step decision-making demands of clinical care [29], [30]. Moreover, commonly used evaluation protocols and metrics often fail to reflect risk-sensitive clinical outcomes. In medical settings, false reassurance and omission errors may lead to asymmetric consequences for patient safety [15], [18], [31]. Real-world deployment further introduces governance constraints, including patient data privacy, auditability, and responsibility allocation between clinicians and AI systems [32], [33]. These gaps highlight the need for a roadmap, which we organize around six closely interconnected challenges: hallucination and factual reliability; evaluation, benchmarks, and clinical validity; safety, ethics, privacy, and regulation; robustness, generalization, and distribution shift; knowledge updating, model maintenance, and lifecycle challenges; and human oversight, workflow integration, and behavior alignment.

Motivated by the rapid advances of Med-LLMs and the persistent gaps between benchmark progress and real-world clinical deployment, this review presents a roadmap for translating Med-LLMs into clinical practice. We synthesize evidence from recent studies using a structured and transparent review approach, organizing the discussion across the development lifecycle of Med-LLMs, including foundational architectures, adaptation strategies, representative applications, evaluation standards, and governance considerations. Given the high-risk nature of medical decision-making, particular attention is devoted to issues essential for safe and reliable clinical adoption, such as hallucination mitigation, risk-aware evaluation, patient privacy protection, auditability, continual knowledge updating, and behavior alignment. The remainder of this paper is organized as follows: 1) describes the search strategy and selection criteria used to identify relevant studies; 2) introduces the foundations of LLMs and summarizes key training and adaptation approaches for Med-LLMs; 3) examines representative applications across major medical domains and their relevance to clinical practice; 4) discusses the main challenges that affect their clinical use, including issues related to reliability and evaluation; 5) outlines future research directions for the development of Med-LLMs, with a focus on clinical validation and real-world deployment; and 6) concludes the review by summarizing the main findings and their implications for clinical practice.

## 2 Search strategy and selection criteria

A structured literature search was conducted using bibliographic databases, including PubMed, Web of Science, Scopus, and the IEEE Xplore Digital Library, supplemented by Google Scholar to cross-check relevant studies and identify additional records. The search focused primarily on peer-reviewed journal articles and major conference proceedings related to Med-LLMs, their clinical applications, multimodal medical AI, evaluation, benchmarking, reliability, and deployment in healthcare settings. Given the rapid development of LLM research, selected influential preprints from arXiv were also considered when they reported important methodological advances, newly released models, or emerging directions not yet fully represented in the peer-reviewed literature. Search terms were selected to capture both methodological and clinical perspectives. A representative search strategy combined LLM-related terms with medical-domain and application-specific terms using Boolean operators. The core search query was: (“large language model” OR “LLM” OR “foundation model” OR “generative artificial intelligence”) AND (“medicine” OR “medical” OR “clinical” OR “healthcare” OR “biomedical”). Additional terms were combined as appropriate to cover major application and challenge areas, including “multimodal medical AI”, “clinical decision support”, “clinical documentation”, “medical question answering”, “mental health”, “telemedicine”, “military medicine”, “evaluation”, “benchmark”, “hallucination”, “reliability”, “safety”, “privacy”, “regulation”, “EU AI Act”, “governance”, “robustness”, “knowledge updating”, and “workflow integration”. The search strategy was adapted according to the syntax and indexing functions of each database. Emphasis was placed on studies published between January 2021 and May 2026, with selected foundational works published before 2021 included where necessary to provide essential background for understanding the development of Med-LLMs.

From the retrieved literature, studies were manually reviewed and selected according to their relevance to the scope of this review. Priority was given to studies with clear clinical relevance, substantial influence in the field, and sufficient methodological or conceptual contribution. Peer-reviewed journal articles and conference papers were prioritized, while influential preprints were included when appropriate to capture recent advances not yet fully represented in the formal literature. Studies that were less relevant to medical contexts, provided limited methodological detail, or substantially overlapped with better-established work were not emphasized. Given the rapid evolution and heterogeneous nature of the literature in this field, rather than conducting a PRISMA-based systematic review or meta-analysis, we used a structured narrative approach to synthesize the selected studies thematically. Focusing on clinical applications, key challenges, and emerging future directions, this strategy aims to provide broad and structured coverage of a rapidly evolving field while maintaining conceptual transparency in how the reviewed evidence was identified and incorporated into the present review.

## 3 Overview of medical large language models

Recent advances in Med-LLMs are fundamentally rooted in architectural and training paradigms developed for general-purpose LLMs [10], [28], [34], [35]. However, applying Med-LLMs in healthcare settings introduces additional requirements related to reliability, safety, data governance, and continual knowledge updating [16], [34], [36], [37]. Accordingly, rather than providing a comprehensive overview of general-purpose LLM development, this section focuses on technical foundations that are most relevant to medical adaptation and trustworthiness [38]. We first summarize core modeling architectures and emerging paradigms, followed by training and adaptation strategies that enable domain specialization, and finally outline technical principles that support the safe and reliable development of Med-LLMs.

### 3.1 Foundations of large language models

LLMs are built upon a combination of architectural innovations and large-scale training paradigms that enable contextual representation and generative reasoning [35], [39]. Understanding these technical foundations is essential for explaining how such models acquire capabilities later adapted to medical applications. Therefore, the key modeling principles underlying contemporary LLMs are examined, including architectural design, scaling behavior, and emerging paradigms, followed by their implications for medical AI and the development of Med-LLMs [40], [41].

#### 3.1.1 Transformer-based architecture

Transformer architectures form the technical foundation of LLMs [35], [42]. Unlike earlier sequence modeling approaches that rely on strictly sequential processing, Transformers employ self-attention mechanisms that enable tokens to interact with other relevant tokens within a given contextual window. In autoregressive decoder-only models, which constitute the dominant architecture for LLMs, attention is typically constrained by causal masking so that predictions depend only on preceding context [9], [12], [43]. This context-aware interaction allows models to capture long-range semantic relationships across extended text, facilitating the integration of dispersed information within complex narratives. Such capabilities are particularly important for medical language processing, where clinically meaningful evidence may appear across multiple sentences, heterogeneous terminologies, or temporally separated clinical descriptions.

In addition to improved contextual representation, Transformer architectures exhibit strong scalability properties that have enabled the development of large-scale foundation models. The attention-based design supports parallel computation during training, allowing efficient optimization on massive datasets compared with recurrent architectures that require sequential updates. As model parameters and training corpora increase, these architectures demonstrate improved generalization across diverse linguistic and knowledge-intensive tasks [10], [43]. This scalability has established Transformers as the dominant backbone for LLMs and has enabled their transfer to specialized domains, including biomedical text analysis, clinical documentation understanding, and medical decision-making scenarios [36].

At the same time, the architectural principles underlying Transformer-based models also influence their behavioral characteristics [44]. Most widely used generative LLMs are trained using autoregressive next-token prediction objectives, producing outputs through probabilistic estimation conditioned on prior context rather than explicit causal reasoning processes. While this mechanism enables flexible language generation and knowledge synthesis, it does not inherently guarantee factual consistency or logical validity [36], [45]. Consequently, both the strengths and limitations of LLMs originate from the same architectural foundation, an observation that becomes particularly relevant when adapting these systems to safety-critical medical applications. Understanding these properties provides an essential basis for subsequent discussions on the development and application of Med-LLMs [40], [46].

#### 3.1.2 Modeling paradigms of large language models

**Fig. 2:**
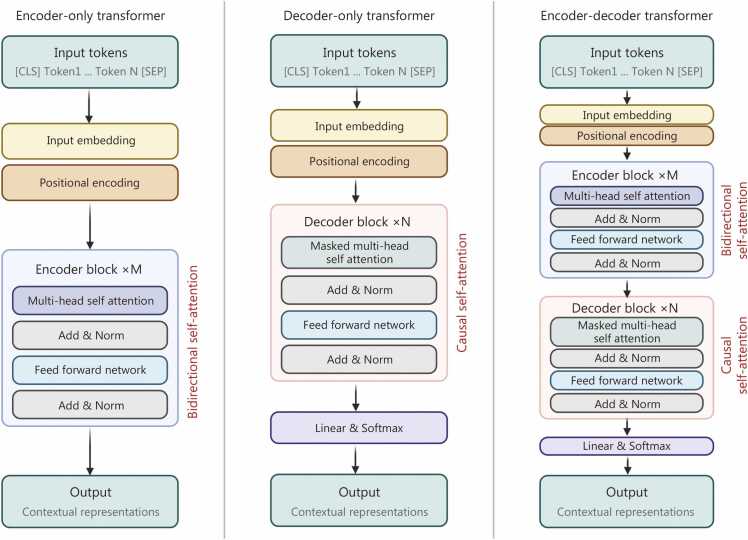
The three basic Transformer architectures: encoder-only Transformer, decoder-only Transformer, and encoder-decoder Transformer. In an encoder-only transformer, input tokens are processed simultaneously through stacked encoder blocks using bidirectional self-attention, allowing each token to attend to all other tokens and produce contextualized representations. In a decoder-only transformer, representations are computed autoregressively from left to right under causal masking, where each token attends only to previously generated tokens for next-token prediction. In an encoder-decoder transformer, source tokens are first encoded into contextual representations by the encoder, and the decoder then generates target representations conditioned on both previous target tokens and the encoder outputs via cross-attention. Arrows indicate the direction of information flow throughout the architectures. CLS. Classification token; SEP. Separator token.

With Transformer architectures serving as the common backbone, LLMs can be broadly categorized into three principal modeling paradigms according to how contextual information is encoded and generated: encoder-only, decoder-only, and encoder-decoder models [7], [47], [48]. These paradigms primarily differ in information flow patterns and learning objectives, which subsequently influence their applicability across downstream tasks [49], [50]. Fig. 2 illustrates the structural characteristics of these modeling paradigms, while Table 1 [8], [9], [11], [12], [13], [43], [51], [52], [53], [54], [55], [56], [57], [58], [59], [60], [61] summarizes representative LLMs associated with each configuration. Rather than representing competing designs, these paradigms provide complementary strategies for language understanding and generation that support a wide range of downstream applications [47], [50].

**Table 1:** Representative Transformer-based language models across encoder-only, decoder-only, and encoder-decoder paradigms.

**Architecture**	**Representative model**	**Year**	**Parameter scale**	**Training data scale**	**Reference**
Encoder-only	BERT	2018	110 M/340 M	3.3 B tokens	[51]
	RoBERTa	2019	355 M	160 GB text	[52]
	DeBERTa	2020	1.5 B	160 GB text	[53]
	ELECTRA	2020	14 M/110 M/335 M	3.3 B tokens	[54]
Decoder-only	GPT-3	2020	175 B	300 B tokens	[55]
	GPT-4	2023	Not reported	Not reported	[43]
	LLaMA	2023	7 B/13 B/33 B/65 B	1.4 T tokens	[8]
	LLaMA 2	2023	7 B/13 B/70 B	2 T tokens	[11]
	LLaMA 3	2024	8 B/70 B	15 T tokens	[12]
	Qwen3	2025	0.6 B–32 B 235 B (22 B active, MoE)	36 T tokens	[9]
	Gemma3	2025	270 M/1 B/4 B/12 B/27 B	Not reported	[56]
	DeepSeek-V3	2024	671 B (37 B active, MoE)	14.8 T tokens	[13]
	GLM-5	2026	744 B (40 B active, MoE)	28.5 T tokens	[57]
Encoder-decoder	BART	2019	140 M/400 M	160 GB text	[58]
	T5	2020	11 B	750 GB text	[59]
	FLAN-T5	2022	3 B/11 B	780 B tokens	[60]
	UL2	2022	20 B	1 T tokens	[61]

Not reported indicates information not specified in the original technical documentation. Parameter values without additional annotation denote dense Transformer models. For MoE architectures, the value outside parentheses represents the total number of model parameters, whereas the value inside parentheses indicates the subset of activated expert parameters involved in computation per token. M. Million; B. Billion; T. Trillion; GB. Gigabyte; MoE. Mixture of experts

*Encoder-only models.* Encoder-only models consist of stacked Transformer encoder layers designed to learn bidirectional contextual representations from input sequences [51], [52]. By simultaneously attending to preceding and succeeding tokens, these models emphasize semantic representation learning rather than open-ended text generation. Such properties are particularly beneficial for medical language understanding tasks requiring precise contextual interpretation, including clinical entity recognition, biomedical information extraction, and electronic health record (EHR) analysis. Early models, including BERT [51] and RoBERTa [52], established the foundation of bidirectional language representation learning, while recent developments such as ModernBERT [62] and domain-adapted variants [63] improve efficiency and long-context modeling for large-scale biomedical text understanding. Despite the rise of generative LLMs, encoder-only models continue to play an important role in medical language understanding pipelines [62], [63], [64].

*Decoder-only models.* Decoder-only models are built upon autoregressive Transformer decoders that generate tokens sequentially under causal attention constraints [65]. This modeling paradigm has become the dominant configuration of LLMs due to its scalability and adaptability across heterogeneous tasks [66]. Through large-scale pretraining on diverse corpora, decoder-only models acquire strong generative capabilities and flexible contextual adaptation, enabling interactive language use, knowledge synthesis, and task generalization without explicit task-specific optimization. Representative foundation model families include GPT [43], [55], Gemini [6], [42], LLaMA [8], [11], [12], Qwen [9], and DeepSeek [7], [13], which increasingly serve as backbones for domain-adapted Med-LLMs. Owing to their capacity for instruction following and in-context adaptation, decoder-only models provide a practical basis for conversational assistance, medical question answering, and clinical documentation support [19], [66], [67].

*Encoder-decoder models.* Encoder-decoder models combine bidirectional input encoding with autoregressive output generation via cross-attention mechanisms, forming a sequence-to-sequence learning framework [59], [61]. This paradigm is particularly suitable for structured transformation tasks requiring faithful mapping between inputs and outputs. In medical contexts, encoder-decoder models remain widely used for clinical report generation, medical summarization, and medical language translation [68]. Beyond the widely used T5 [59] and instruction-tuned FLAN-T5 [60], encoder-decoder variants such as UL2 [61] and FLAN-UL2 further strengthen generalization through unified pretraining and instruction tuning, while LongT5 [69] extends the framework to longer clinical documents. This advantage is also consistent with evidence from general sequence-to-sequence evaluations, where representative models such as T5 and UL2 have shown strong performance on summarization, translation, and other text-to-text benchmarks [59], [60], [61]. Such properties make them particularly suitable for medical tasks that require faithful transformation from clinical inputs to structured or narrative outputs. In the biomedical domain, encoder-decoder adaptations including BioBART [70] and SciFive [71] are frequently used backbones for generation-oriented biomedical NLP tasks.

The coexistence of multiple modeling paradigms reflects the heterogeneous requirements of medical AI applications [72]. Encoder-only models emphasize reliable semantic representation, decoder-only models enable flexible knowledge synthesis and interaction, and encoder-decoder models facilitate structured information transformation. These distinctions influence task suitability as well as controllability and robustness considerations when adapting LLMs to safety-critical domains [38], [40]. Understanding paradigm-level differences, therefore, provides an essential foundation for analyzing the development and application of Med-LLMs discussed in subsequent sections [19].

#### 3.1.3 Scaling and emergent capabilities

A central empirical observation in the development of LLMs is that model performance often improves predictably with scale [39], [73]. Prior work has described power-law relationships between training loss and key scaling factors, including model parameters, dataset size, and training compute, suggesting that better performance can be achieved by increasing resources under a fixed optimization recipe [10], [74]. Subsequent studies further emphasized that compute-optimal training depends on jointly scaling model size and the amount of training data, rather than expanding parameters alone, highlighting the practical role of data and compute allocation in shaping achievable capability at a given budget [75], [76].

As scaling progresses, LLMs can exhibit qualitative capability shifts on certain tasks, sometimes described as emergent behaviors [77]. These behaviors may include improved multi-step problem solving and reasoning-like patterns that are not evident at smaller scales, although their onset and measurability can depend on task design and evaluation granularity [77], [78]. In parallel, instruction following is largely enabled by post-training alignment processes, such as supervised instruction tuning and preference-based optimization with human feedback, which substantially improve helpfulness and intent-following relative to base next-token predictors [79], [80], [81]. Scaling and instruction alignment do not necessarily guarantee reliability in safety-critical domains [38], [40]. LLMs remain probabilistic systems and may produce unstable or incorrect outputs when facing distribution shifts, ambiguous inputs, or incomplete clinical information. Improvements observed on benchmark evaluations, therefore, do not directly translate into reliable real-world medical performance [31]. These limitations highlight the need for evaluation frameworks and governance mechanisms specifically designed for risk-sensitive healthcare applications [37], [46], [82].

### 3.2 Emerging directions in large language models design

As LLMs are scaled to support broader capabilities and longer contexts, attention-based dense Transformers face practical constraints in compute, memory, and knowledge updating. In response, several design directions have gained momentum that complement standard dense Transformer scaling, with particular relevance to safety-critical domains such as medicine [83], [84].

Recent research has explored alternative sequence modeling approaches that reduce reliance on full self-attention mechanisms [85]. Among these, state-space models (SSMs) have emerged as a promising direction for efficient long-sequence modeling [86], [87]. Instead of explicitly computing pairwise attention across tokens, SSMs model sequences through recurrent state transitions, enabling computational costs that scale more favorably with input length. Selective state-space architectures such as Mamba demonstrate competitive language modeling performance while improving efficiency in long-context processing. These properties are particularly relevant for domains involving extended textual or temporal information, where maintaining long-range dependencies under practical computational constraints remains challenging. Although SSM-based models are still under active investigation and have not replaced attention-based architectures, they represent an important complementary direction for improving scalability and efficiency in next-generation LLM design [88].

Another major direction is to decouple part of factual knowledge from model parameters by integrating non-parametric memory, typically via retrieval [15]. RAG combines a retriever with a generator to condition outputs on retrieved evidence for knowledge-intensive tasks [15], [89]. Retrieval-Enhanced Transformer (RETRO) further illustrates retrieval integrated into the modeling pipeline, reporting competitive performance with substantially fewer parameters by conditioning generation on retrieved chunks from a large corpus [90]. For Med-LLMs, retrieval-integrated designs provide a principled mechanism to ground responses in updatable sources such as guidelines and institutional knowledge bases, which can improve traceability and support governance requirements when combined with appropriate evaluation and auditing [91], [92].

MoE architectures introduce sparsity into LLMs by activating only a portion of model parameters during each computation step [14]. This design allows model capacity to scale substantially while maintaining manageable computational cost, and it has been adopted by several recent high-performance LLMs [14], [93]. Sparse activation improves computational efficiency under practical resource constraints, which is relevant for the adaptation of Med-LLMs in real-world settings. However, increased system complexity may introduce challenges in optimization stability and deployment management [94]. Consequently, while MoE improves scalability, it does not inherently address reliability or safety requirements in medical applications.

These developments suggest that advances in LLM capability are no longer driven solely by increases in model scale [87], [92], [93]. Emerging design strategies emphasize computational efficiency, improved handling of long-context information, and closer integration with external knowledge sources. Such directions provide important technical foundations for improving adaptability and controllability in Med-LLMs, particularly in environments where computational resources and knowledge updating remain practical concerns [95], [96]. Nevertheless, architectural innovation alone cannot ensure reliable behavior in medical settings. Further progress is needed in evaluation, alignment, and governance frameworks, as discussed in subsequent sections of this review.

### 3.3 Domain requirements for medical large language models

Although general-purpose LLMs are trained on web-scale corpora and show strong language competence, their direct transfer to medicine is limited by a pronounced domain shift [83], [97]. Clinical text differs from general-domain language in vocabulary and style, with dense jargon, abbreviations, idiosyncratic documentation practices, and mixed structured and free-text formats [98], [99]. These characteristics make clinical narratives harder to model reliably than general public-domain text and can degrade extraction and understanding when models are not adapted to clinical distributions. Beyond language mismatch, medical knowledge and clinical reasoning impose distinct constraints [40]. Clinical decisions often require multi-step inference under uncertainty, reconciliation of incomplete or noisy evidence, and risk-sensitive judgment where different error types have asymmetric consequences for patient safety and resource allocation. As a result, improvements on general benchmarks or in fluent generation do not necessarily translate into safe clinical behavior, especially when models encounter ambiguous presentations or out-of-distribution cases [38], [100].

Medical applications operate in safety-critical contexts where model errors may directly affect clinical decisions and patient outcomes [84]. Strict privacy protection and data governance frameworks limit the collection, sharing, and secondary use of medical data, thereby constraining large-scale model training and evaluation [101]. At the same time, medical knowledge evolves continuously as new evidence and clinical guidelines emerge, requiring mechanisms for controlled updating and traceable knowledge integration. These characteristics distinguish medical settings from general language modeling environments and place additional demands on reliability, transparency, and accountable model behavior in the development of Med-LLMs [37], [102].

### 3.4 Training and adaptation strategies for medical large language models

Med-LLMs are typically developed by adapting general-purpose LLMs to address the linguistic, knowledge, and safety constraints of medical domains [26], [83]. This adaptation does not rely on a single modification step but instead involves a coordinated set of training and alignment strategies. Collectively, these approaches aim to reduce domain mismatch, guide clinically appropriate model behavior, enable resource-aware customization, and support knowledge grounding through external evidence [91], [103], [104]. Representative technical strategies for Med-LLM training and adaptation are summarized in Table 2 [16], [21], [22], [24], [25], [26], [105], [106], [107], [108], [109], [110], [111], [112], [113], [114], [115], [116], [117], [118], [119], [120], [121], [122], [123].

**Table 2:** Representative technical strategies for medical large language models (Med-LLMs) training and adaptation.

**Category**	**Model**	**Year**	**Backbone**	**Modality**	**Adaptation method**	**Medical data type**	**Reference**
Continued pretraining	BioGPT	2022	GPT-style LM	Text	Domain-adaptive pretraining	Biomedical literature	[105]
	GatorTron	2022	Transformer LM	Text	Domain-adaptive pretraining	Clinical text and biomedical literature	[106]
	GatorTronGPT	2023	GPT-style LM	Text	General domain pretraining	Clinical corpora	[107]
	PMC-LLaMA	2023	LLaMA	Text	Continued pretraining	Biomedical literature	[21]
	MEDITRON	2023	LLaMA-2	Text	Continued pretraining	Biomedical literature and guidelines	[108]
	BioMedLM	2024	GPT-style LM	Text	Domain-adaptive pretraining	Biomedical literature	[109]
Supervised fine-tuning	ChatDoctor	2023	LLaMA	Text	Dialogue instruction tuning	Clinical consultations	[24]
	MedAlpaca	2023	LLaMA	Text	Instruction tuning	Medical QA data	[25]
	HuatuoGPT	2023	General LLM	Text	Instruction tuning	Clinical consultations	[110]
	ClinicalGPT	2023	General LLM	Text	Task-specific fine-tuning	Clinical text and consultations	[111]
	Med-PaLM 2	2023	PaLM-2	Text	Instruction tuning	Medical QA data	[16]
	LLaVA-Med	2023	Vision-language model	Multimodal	Multimodal instruction tuning	Medical image-text pairs	[112]
	MAIRA-2	2024	Vision-language model	Multimodal	Supervised report alignment	Chest X-ray reports	[113]
Parameter-efficient adaptation	Clinical Camel	2023	LLaMA-2	Text	QLoRA	Clinical dialogue	[22]
	MedAdapter	2023	BERT	Text	Adapter tuning	Biomedical literature	[114]
	Med42	2024	LLaMA-2	Text	PEFT	Medical benchmark	[115]
	MedLoRA	2024	LLaMA	Text	LoRA	Medical instruction	[116]
	Me-LLaMA	2025	LLaMA-2	Text	LoRA	Medical QA data	[26]
	PeFoMed	2024	Vision-language model	Multimodal	Multimodal PEFT	Medical image-text pairs	[117]
	AnyRef	2024	Vision-language model	Multimodal	Multimodal LoRA	Medical image-text pairs	[118]
Retrieval-augmented generation	Almanac	2024	GPT-4	Text	Guideline-grounded retrieval	Clinical guidelines	[119]
	MedRAG	2025	Multiple LLMs	Text	Evidence-grounded retrieval	Biomedical literature	[120]
	ClinicalRAG	2024	Multiple LLMs	Text	EHR-grounded retrieval	Clinical records	[121]
	i-MedRAG	2024	Multiple LLMs	Text	Iterative retrieval	Biomedical literature	[122]
	Omni-RAG	2025	Multiple LLMs	Multimodal	Multimodal RAG	Medical image-text pairs	[123]

Multiple LLMs indicates that the framework can operate with different underlying LLMs (e.g., GPT series, LLaMA series, or other general-purpose LLMs), depending on deployment configuration. Vision-language model refers to a multimodal model jointly trained to process visual and textual inputs and to learn aligned representations across image and text modalities.EHR. Electronic health record; KG. Knowledge graph; LM. Language model; LLM. Large language model; MoE. Mixture of experts; PEFT. Parameter-efficient fine-tuning; QA. Question answering; QLoRA. Quantized low-rank adaptation; RAG. Retrieval-augmented generation

A common starting point is domain-adaptive or continued pretraining on biomedical and clinical corpora [26], [104], [124]. This stage mitigates distributional differences between general web-based text and medical language by exposing the model to domain-specific terminology, documentation patterns, and contextual semantics [108]. Although the core objective typically remains language modeling, the shift in training data improves representation quality for medical concepts and discourse. Continued pretraining has therefore become a practical and widely adopted approach for medical adaptation, particularly when training LLMs from scratch is constrained by data access and computational cost [26], [83], [108].

Following domain adaptation, SFT and instruction-based alignment are applied to shape how models respond in medical contexts [125]. SFT leverages curated medical tasks or instruction-formatted data to improve output relevance, structure, and consistency. Instruction tuning enhances the model’s ability to follow clinically meaningful prompts, including appropriate expression of uncertainty and adherence to requested output formats [104], [126]. In addition, preference-based alignment methods are increasingly used to constrain undesirable outputs and promote more consistent behavior under complex or ambiguous prompts [80]. In medical settings, these alignment stages serve to bound model behavior within clinically acceptable expectations rather than merely improving task performance [103], [125]. To address practical constraints in computation and governance, PEFT methods are often employed [127]. Instead of updating all parameters of a large model, PEFT approaches modify only a small subset while keeping the majority of the base model fixed. This strategy substantially reduces memory and training costs, facilitating iterative adaptation across institutions, specialties, or evolving clinical subdomains [128], [129]. This efficiency is especially important in medical contexts where data governance constraints and resource limitations make repeated full-parameter fine-tuning impractical.

Because medical knowledge evolves and clinical recommendations are periodically updated, many Med-LLMs incorporate RAG and related knowledge integration mechanisms [91], [95]. Instead of relying only on knowledge stored in model parameters, RAG-based approaches enable Med-LLMs to retrieve relevant information from external sources, such as clinical guidelines, biomedical literature, or institutional knowledge bases, during response generation. This design can improve traceability and make outputs easier to update when medical evidence changes [15]. Retrieval-based grounding does not eliminate all potential failure modes. However, it can improve factual consistency and help align responses with current clinical references when supported by appropriate source control and validation procedures [92], [95]. Continued pretraining, behavioral alignment, parameter-efficient adaptation, and retrieval-based grounding together define the primary technical pathways through which general LLMs are transformed into Med-LLMs. Each pathway addresses distinct domain-specific constraints, including distributional mismatch, behavioral control, resource limitations, and knowledge updating. However, these strategies function as complementary components rather than standalone solutions, and their effectiveness ultimately depends on careful integration and context-sensitive evaluation [83], [125], [130].

### 3.5 Principles for medical large language models

The previous sections outlined how Med-LLMs develop capability through model architecture and domain adaptation [31], [84]. However, stronger model performance does not necessarily translate into reliable behavior in medical settings [131]. The requirement for trustworthiness arises from intrinsic characteristics of current LLM training paradigms and evaluation practices. Most contemporary LLMs are optimized for next-token prediction, a learning objective that favors statistical coherence rather than calibrated factual correctness or evidence-based reasoning [132]. In medical settings, where inputs are often incomplete or uncertain, this optimization can produce plausible yet unsupported outputs [133]. At the same time, commonly used evaluation benchmarks rely on static datasets and aggregate accuracy metrics, which may inadequately capture asymmetric clinical risks or context-dependent decision pathways [31], [134].

These challenges are further shaped by structural properties of medical data [135]. Clinical information is sensitive, institution-specific, and heterogeneous across populations and documentation standards [136], [137]. These constraints limit large-scale data sharing and introduce distributional variability between training and deployment environments. As a result, Med-LLMs may encounter domain shifts or incomplete coverage of rare conditions, complicating reliability assessment and cross-institutional generalization [138]. Behavioral alignment introduces additional complexity. Clinical recommendations are context-dependent and embedded within responsibility structures that vary across specialties and operational settings [131]. Alignment signals obtained during training cannot fully represent this variability, and acceptable risk thresholds may differ across use cases. Consequently, the need for human oversight and boundary-setting emerges from the institutional nature of medical decision-making rather than from model capability alone [82].

Finally, the dynamic evolution of medical knowledge creates a structural tension between static model parameters and continuously updated clinical evidence [134]. Guidelines, therapeutic standards, and regulatory frameworks change over time, whereas model representations remain fixed unless explicitly updated. This temporal mismatch makes continual knowledge updating a foundational concern. Taken together, these factors demonstrate that the performance and deployment of Med-LLMs are shaped by training objectives, evaluation assumptions, governance constraints, alignment processes, and knowledge dynamics [139]. Building on this foundation, the following section examines how these structural characteristics manifest across diverse medical applications.

## 4 Applications of medical large language models

**Fig. 3:**
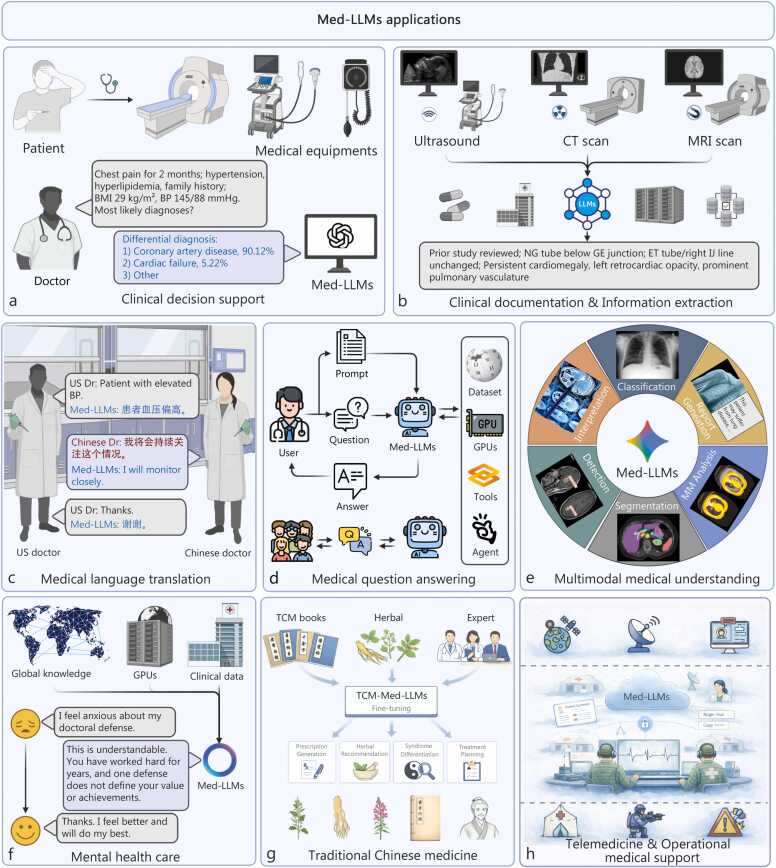
Comprehensive overview of potential applications of large language models in the medical domain. **a** Clinical decision support. **b** Clinical documentation and information extraction. **c** Medical language translation. **d** Medical question answering. **e** Multimodal medical understanding. **f** Mental health care. **g** Traditional Chinese medicine. **h** Telemedicine and operational medical support. BMI. Body mass index; BP. Blood pressure; CT. Computed tomography; ET tube. Endotracheal tube; GE junction. Gastroesophageal junction; IJ. Internal jugular; LLM. Large language model; Med-LLM. Medical large language model; MRI. Magnetic resonance imaging; NG tube. Nasogastric tube; TCM. Traditional Chinese medicine.

Recent progress in Med-LLMs has stimulated growing interest in their potential roles across a range of medical and clinical tasks. Building on the literature identified through the review methodology described in Section 2 (**Search strategy and selection criteria**), this section examines several key application domains of Med-LLMs, including clinical decision support, medical documentation, question answering, multimodal medical data analysis, medical translation, mental health care, and traditional Chinese medicine (TCM). To provide a coherent overview, the discussion is organized around major categories of medical tasks that broadly reflect common clinical and healthcare workflows. Within each domain, we examine how Med-LLMs are used in practice and summarize the data sources and evaluation approaches reported in existing studies. We also highlight practical factors that may affect their adoption in clinical or healthcare environments. Rather than cataloguing individual datasets or systems, the goal is to identify recurring patterns in how these models are integrated into different medical contexts. Fig. 3 presents a comprehensive overview of major application scenarios of Med-LLMs, which will be discussed in detail in the following sections. Table 3 [16], [140], [141], [142], [143], [144], [145], [146], [147], [148], [149], [150], [151] and Table 4 [26], [28], [66], [68], [106], [112], [120], [152], [153], [154], [155], [156], [157], [158], [159], [160], [161], [162], [163], [164], [165], [166], [167], [168], [169], [170], [171], [172], [173], [174], [175], [176] provide an overview of representative datasets, benchmark tasks, and evaluation metrics for Med-LLM development and assessment, as well as their applications across major clinical domains.

**Table 3:** Representative datasets, benchmark tasks, and evaluation metrics for medical large language models (Med-LLMs) development and assessment.

**Dataset**	**Year**	**Modality**	**Task category**	**Data scale**	**Evaluation metrics**	**Capability assessed**	**Reference**
MedQA	2020	Text	Medical QA, exam reasoning	61,097 questions	Accuracy	Medical reasoning	[140]
PubMedQA	2019	Text	Biomedical evidence inference	1000 labeled questions; 61,200 unlabeled instances; 211,300 artificial instances	Accuracy, F1-score	Medical evidence interpretation	[141]
MedMCQA	2022	Text	Medical QA, exam reasoning	194,000 questions	Accuracy	Medical reasoning	[142]
MultiMedQA	2023	Text	Medical QA, exam reasoning	7 integrated QA datasets	Accuracy	Clinical reasoning	[16]
MIMIC-III	2016	Text, tabular	Clinical prediction, risk modeling	58,000 hospital admissions	AUROC, AUPRC, F1-score, C-index	Clinical risk prediction	[143]
UK Biobank	2015	Image, tabular	Clinical prediction, risk modeling	502,616 participants	AUROC, AUPRC, F1-score, C-index	Population risk prediction	[144]
MIMIC-CXR	2019	Multimodal	Report generation	377,110 images; 227,835 studies	BLEU, ROUGE, METEOR, BERTScore, BARTScore, RadGraph F1, CheXbert F1	Radiology report generation	[145]
IU X-Ray	2016	Multimodal	Report generation	7470 images; 3955 reports	BLEU, ROUGE, METEOR, BERTScore, BARTScore	Radiology report generation	[146]
CheXpert	2019	Image	Image classification, label prediction	224,316 images; 65,240 patients	AUROC, AUPRC, F1-score, sensitivity, specificity	Disease classification	[147]
CXPMRG	2025	Multimodal	Report generation	223,462 image-report pairs; 187,711 studies	BLEU, BERTScore, RadGraph F1, CheXbert F1, RadCliQ	Radiology report generation	[148]
VQA-RAD	2018	Multimodal	Medical VQA	315 images; 3515 QA pairs	Accuracy, F1-score	Radiology vision-language reasoning	[149]
PathVQA	2020	Multimodal	Medical VQA	4998 images; 32,799 QA pairs	Accuracy, F1-score	Pathology vision-language reasoning	[150]
OmniMedVQA	2024	Multimodal	Medical VQA	73 source datasets; 12 modalities; 20 anatomical regions	Accuracy, F1-score	General medical vision-language reasoning	[151]

AUPRC. Area under the precision-recall curve; AUROC. Area under the receiver operating characteristic curve; BLEU. Bilingual evaluation understudy; C-index. Concordance index; METEOR. Metric for evaluation of translation with explicit ordering; QA. Question answering; ROUGE. Recall-oriented understudy for gisting evaluation; VQA. Visual question answering.

**Table 4:** Representative medical large language models (Med-LLMs) applications in major clinical domains, together with their development methods, medical tasks, and clinical roles.

**Application**	**Model**	**Year**	**Modality**	**Method**	**Medical task**	**Clinical role**	**Reference**
Clinical decision support	GatorTron	2022	Text	Clinical-domain pretraining	Clinical text understanding	EHR-based decision support	[106]
	TrialGPT	2024	Text	RAG	Trial eligibility assessment	Clinical trial matching	[152]
	Med-PaLM 2	2025	Text	Instruction tuning	Medical question answering	Diagnostic decision-making	[66]
	ClinicalGPT-R1	2025	Text	RL	Diagnostic reasoning	General clinical decision support	[153]
Clinical documentation & extraction	ACS-LLM	2024	Text	PEFT	Clinical text summarization	Clinical note drafting	[68]
	EHR-Tools	2025	Text/EHR	Prompt ensemble	Hospital course generation	Workflow-integrated documentation support	[154]
	Flamingo-CXR	2025	Multimodal	Multimodal fine-tuning	Radiology report generation	Radiology documentation drafting	[155]
	CLEAR	2025	Text	RAG	Clinical information extraction	Structured clinical entity extraction	[156]
Medical language translation	MMed-Llama3	2024	Text	Continued pretraining	Multilingual medical translation	Multilingual medical communication	[157]
	LLMs-in-MT	2024	Text	PEFT	Biomedical text translation	Domain-specific translation	[158]
	MedCOD	2025	Text	LoRA	English-to-Spanish medical translation	Terminology-preserving translation	[159]
	MultiMed-ST	2025	Speech/Text	Instruction tuning	Medical speech translation	Cross-lingual clinical communication	[160]
Medical question answering	LLaVA-Med	2023	Multimodal	Multimodal instruction tuning	Biomedical visual question answering	Biomedical image interpretation	[112]
	Me-LLaMA	2024	Text	Continued pretraining	Biomedical question answering	Biomedical knowledge retrieval assistance	[26]
	MedRAG	2024	Text	RAG	Evidence-grounded question answering	Medical evidence retrieval and answering	[120]
	LINS	2025	Text	Multi-agent RAG	Evidence-grounded question answering	Credible evidence-based answering	[161]
Multimodal medical understanding	scGPT	2024	Multimodal	Multimodal pretraining	Multi-omics representation learning	Molecular phenotype characterization	[162]
	MedGemma	2025	Multimodal	Multimodal pretraining	Medical image-text understanding	Open medical model development	[28]
	HONeYBEE	2025	Multimodal	Foundation-model embeddings	Multimodal oncology representation learning	Patient-level oncology profiling	[163]
	MOFS	2025	Multimodal	Intermediate & late multimodal fusion	Multi-omics subtype discovery	Precision glioma stratification	[164]
Mental health care	MentaLLaMA	2024	Text	Instruction tuning	Depression risk detection	Social-media mental health screening	[165]
	CBT-LLM	2024	Text	Instruction tuning	Mental health question answering	CBT-aligned psychoeducational dialogue	[166]
	MLlm-DR	2026	Multimodal	PEFT	Depression severity assessment	Multimodal mental health evaluation	[167]
	MDD-Thinker	2026	Text	PEFT & RL	Clinical depression diagnosis reasoning	Diagnostic decision support	[168]
Traditional Chinese medicine	Qibo	2024	Text	Instruction tuning	TCM question answering	TCM knowledge consultation	[169]
	JingFang	2025	Text	Multi-agent reasoning	Syndrome differentiation and treatment recommendation	TCM knowledge consultation	[170]
	ShizhenGPT	2025	Multimodal	Multimodal pretraining	Image-text TCM understanding	Multimodal TCM interpretation	[171]
	TCM-KLLaMA	2025	Text	Knowledge graph integration	Herbal prescription generation	Prescription planning	[172]
Telemedicine and operational medical support	ED Handoff LLM	2024	Text	Prompt engineering	Handoff note generation	Transition-of-care communication	[173]
	GPT-4o TCCC	2025	Text	Guideline-grounded prompting	Ventilator management decision support	Battlefield casualty management	[174]
	MedAgentBench	2025	Text/EHR	LLM-agent orchestration	Longitudinal EHR task execution	Operational workflow automation	[175]
	SELSM-Agent	2026	Text/EHR	Retrieval-guided agent reasoning	FHIR-based clinical decision tasks	Edge-deployable medical agent support	[176]

CBT. Cognitive behavioral therapy; ED. Emergency department; EHR. Electronic health record; FHIR. Fast Healthcare Interoperability Resources; LLM. Large language model; LoRA. Low-rank adaptation; MDD. Major depressive disorder; MT. Machine translation; PEFT. Parameter-efficient fine-tuning; QA. Question answering; RAG. Retrieval-augmented generation; RL. Reinforcement learning; ST. Speech translation; TCCC. Tactical combat casualty care; TCM. Traditional Chinese medicine.

### 4.1 Clinical decision support

Clinical decision support refers to systems designed to assist clinicians in synthesizing patient information and supporting diagnostic reasoning, rather than replacing medical judgment [40], [84]. In clinical practice, physicians must integrate heterogeneous evidence, including symptoms, laboratory results, medical history, and imaging findings, often under considerable time pressure and uncertainty [20], [177]. Med-LLMs have recently been explored as tools capable of interpreting clinical narratives, summarizing longitudinal patient records, and proposing differential diagnostic hypotheses based on available information [154], [178]. Unlike earlier rule-based systems, these models can process unstructured medical language and capture contextual relationships within patient records. This capability makes them potentially useful for tasks such as triage support, diagnostic reasoning assistance, and risk stratification. At the same time, these applications highlight the importance of reliability and interpretability, because even seemingly plausible suggestions may influence clinical judgment if presented without appropriate context [40], [179].

Med-LLMs are commonly incorporated into clinical decision support through two complementary patterns. In the conversational setting, a model supports case discussion by producing structured summaries, identifying missing or conflicting information, and outlining plausible diagnostic explanations in response to clinician queries [180]. In parallel, many systems couple Med-LLMs with external medical knowledge sources such as clinical guidelines, curated databases, or local institutional resources, so that outputs can be grounded in retrievable evidence rather than relying solely on parametric memory [181]. RAG is frequently used in this evidence-grounded setting to facilitate traceable synthesis and reduce unsupported statements, especially when recommendations depend on up-to-date or institution-specific guidance [91], [182]. Beyond free-form dialogue, recent work has also explored more structured workflows in which intermediate decisions are made explicit and can be audited [152], [181]. TrialGPT illustrates this direction by parsing eligibility criteria, assessing criterion-level patient eligibility, and ranking candidate trials to support clinical trial matching [152]. Together, these developments show how Med-LLMs can function not only as conversational assistants, but also as evidence-aware components within workflow-oriented decision support.

Evidence for Med-LLMs’ decision support is currently derived from two main forms of evaluation. One approach relies on benchmark-style datasets that assess medical knowledge and reasoning ability through structured questions, including multiple-choice examinations and curated question-answering tasks [66], [183]. Evaluations such as MultiMedQA [16] and MedQA [140] have been widely used to measure whether models can retrieve and apply biomedical knowledge in controlled settings. The second approach focuses on tasks derived from clinical documentation, where the goal is to assess whether generated outputs are clinically meaningful and safe in practical workflows [184]. Studies in emergency medicine and other clinical contexts have examined tasks such as summarizing EHRs or assisting with clinical handoffs [173], [185], [186]. In these settings, evaluation often emphasizes usefulness, clarity, and safety rather than accuracy alone [187]. The choice of evaluation metrics also depends on the clinical decision support task. In structured prediction tasks such as triage support, risk stratification, or diagnostic classification, model performance is commonly evaluated using metrics such as precision, recall, and F1 score, which help characterize different types of diagnostic errors and their potential clinical implications [143], [147], [148]. For example, high recall may be particularly important when the goal is to avoid missing high-risk patients, whereas low precision may increase unnecessary alerts or downstream review burden. In documentation-oriented tasks such as clinical summarization or handoff support, fluent output alone is insufficient, because omission of critical findings or medications may reduce clinical safety even when the generated text appears coherent. In such settings, factual consistency, completeness, and clinician judgment are often needed alongside conventional performance metrics to assess whether outputs are truly useful in practice. Together, these two evaluation paradigms provide complementary perspectives, although neither fully captures the complexity of real clinical decision-making.

Despite these promising developments, the deployment of Med-LLMs in clinical decision support faces several practical constraints. Clinical data vary substantially across institutions, specialties, and documentation practices, which can introduce distribution shifts that affect model reliability [136], [185]. The probabilistic nature of language generation also means that confident but unsupported statements may occasionally appear in outputs, creating potential risks if results are interpreted without verification [20]. In addition, healthcare environments impose strict requirements related to privacy protection, data governance, and accountability. Models must therefore operate within regulatory and institutional frameworks that limit data sharing and require traceability of system behavior [152]. Clinical knowledge also evolves continuously as new evidence emerges and treatment guidelines change. Maintaining the relevance of Med-LLM systems in such environments requires mechanisms for updating knowledge and monitoring model behavior over time. These considerations illustrate why evaluating Med-LLMs’ decision support requires not only performance metrics but also careful attention to integration within clinical workflows [20], [84], [188].

### 4.2 Clinical documentation and information extraction

Clinical documentation is a fundamental part of clinical practice, but it is also widely recognized as a major source of administrative workload for clinicians [136], [185]. Recent research suggests that Med-LLMs can assist with drafting and revising clinical text while also helping transform narrative records into structured information that can be used for care coordination, billing, and research activities [189], [190]. In this section, clinical documentation is considered in a broad sense. It includes the generation of narrative reports such as discharge summaries and procedure notes, as well as the extraction and normalization of information from free-text clinical records [156], [186]. These processes are closely connected in routine practice. Clinical notes are often created from multiple sources of information and later converted into structured elements such as problem lists, medication histories, allergy records, and diagnostic codes [128], [191]. Considering documentation from this workflow perspective helps explain why reliability and safety are particularly important. Errors in documentation may not only affect the medical record itself but may also influence clinical decisions, quality reporting, and administrative processes [192].

Across the literature, Med-LLMs are incorporated into clinical documentation workflows through both generation-oriented and structuring-oriented designs, and these approaches are often combined in practice. In generation-oriented settings, models assist in drafting clinical narratives from encounter information, including discharge summaries, progress notes, procedure notes, and radiology reports [185], [186], [193]. These systems aim to reduce the time clinicians spend composing routine documentation while helping organize key findings and summarize patient encounters in a clearer narrative form. In most proposed deployments, the generated text is reviewed and edited by clinicians rather than used directly, and the models function primarily as drafting assistants instead of fully autonomous documentation systems [194]. Generating clinical documentation also presents distinct challenges, because notes must remain faithful to the underlying patient record, reflect diagnostic uncertainty when appropriate, and avoid introducing unsupported information [186], [195]. Alongside narrative drafting, Med-LLMs are increasingly used to extract clinically relevant information from free-text records and convert it into structured elements such as problem lists, medication histories, and allergy records. These structured outputs can support downstream tasks, including coding, cohort identification, and clinical research data preparation [191], [196]. In practice, documentation generation and information extraction are often integrated within the same workflow, allowing narrative notes to be produced while key information is simultaneously organized into structured representations that can be reused across clinical systems [41].

Evaluation of documentation-oriented Med-LLMs commonly blends automatic metrics with expert judgment, reflecting the gap between text similarity and clinical usefulness [197]. For report generation, overlap metrics such as Recall-Oriented Understudy for Gisting Evaluation (ROUGE) [198] and Bilingual Evaluation Understudy (BLEU) [199] remain common, but many studies emphasize that high overlap does not guarantee factual correctness, appropriate omissions, or safe phrasing in clinical contexts [193], [195], [200]. As a result, human evaluation is frequently used to assess correctness, completeness, and utility, often with clinician raters and workflow-relevant rubrics. For information extraction, standard measures such as Precision, Recall, and F1 score are widely used because outputs can be compared against annotated spans or structured labels [190], [200]. Coding support and administrative applications often use accuracy and error analysis across large label spaces, where performance can be sensitive to note noise and local coding conventions [191]. Evidence increasingly points to the need for task-specific evaluation that distinguishes harmless stylistic variation from clinically meaningful errors.

Several practical constraints influence whether these systems can be used safely in real clinical environments [84], [201]. First, generated documentation may contain unsupported additions or subtle distortions. This issue becomes particularly problematic when clinical notes are treated as authoritative records. As a result, many studies emphasize cautious deployment with clear clinician oversight and traceability [186], [194], [197], [200]. Second, privacy and governance requirements limit data sharing and logging. These constraints also make model improvement more difficult because training signals are embedded in protected health information [136]. Third, documentation practices vary across institutions, specialties, and EHR systems. Such variation can create distribution shifts that reduce reliability when systems are transferred without local validation. Finally, administrative tasks such as medical coding illustrate that fluent text generation does not necessarily translate into reliable structured outputs. Empirical studies have shown that general LLMs may perform poorly in medical coding scenarios [189], [191], [196]. This observation highlights the importance of domain constraints and careful evaluation before these systems are used in operational settings. These issues motivate the trustworthiness challenges discussed later in this review, particularly those related to hallucination, evaluation, and governance [201].

### 4.3 Medical language translation

Medical language translation plays an important role in clinical communication across linguistic and cultural boundaries [202], [203]. In healthcare settings, it involves not only transferring medical information between languages but also reformulating professional medical expressions into forms that can be more easily understood by different audiences. Such tasks arise in multilingual clinical encounters, informed consent, discharge communication, and the international exchange of medical knowledge [204]. In these contexts, the primary requirement is not only linguistic fluency but also the preservation of clinical meaning [203], [205]. A grammatically correct translation may still distort terminology, omit uncertainty, or alter the practical implications of the original medical statement. For this reason, recent studies increasingly emphasize communication quality, clarity, and safety when evaluating language conversion in medical contexts [205], [206].

Current Med-LLM applications in this area broadly follow several patterns. The first involves clinician-facing translation, where the goal is to preserve technical precision in multilingual clinical records, guideline interpretation, or cross-institutional evidence exchange [157], [207]. A second scenario involves translation and reformulation aimed at patient communication, where complex medical language is converted into clearer and more accessible explanations for non-expert audiences [202]. A third direction focuses on translation grounded in external medical knowledge sources, where terminology systems, curated references, or biomedical literature are incorporated to improve semantic fidelity [206]. Recent multilingual medical foundation models illustrate this trend. For example, MMed-Llama3 [157] was developed together with a large multilingual medical corpus and benchmark, reflecting a shift toward multilingual medical language modeling rather than English-centric adaptation alone. Other recent work, such as MedCOD [159], suggests that incorporating structured medical knowledge can further improve translation accuracy in specialized biomedical contexts.

The data and evaluation landscape for medical language translation remains narrower than the communication demands encountered in real clinical environments [203], [207]. Classic biomedical translation resources, such as the European Medicines Agency (EMEA) corpus [208], continue to provide widely used multilingual medical text for training and evaluation [209]. In addition, the Conference on Machine Translation (WMT) Biomedical Translation Task offers standardized benchmarks for assessing translation performance across biomedical language pairs. More recent multilingual medical LLM research has also introduced broader evaluation resources, such as MMedBench, which assesses multilingual medical understanding across multiple tasks rather than focusing solely on sentence-level translation [157]. Evaluation typically relies on automatic metrics such as BLEU together with neural evaluation metrics like Crosslingual Optimized Metric for Evaluation of Translation (COMET) [210]. However, these metrics primarily measure textual similarity and may not fully capture terminology accuracy, preservation of clinical meaning, or the appropriateness of translated content for clinical use. For this reason, expert review remains particularly important in medical contexts, where even small translation errors can alter clinical interpretation [203].

Several practical limitations continue to constrain the use of Med-LLMs for medical language translation [204]. Medical terminology is highly context-dependent, and errors involving drug names, procedures, abbreviations, or risk statements may alter clinical meaning even when the translated text appears fluent [159], [205]. Translation and reformulation for patient-facing communication introduce an additional tension between readability and fidelity, because simplification can improve comprehension while also removing nuance or uncertainty. Multilingual imbalance remains another concern, as English-dominant training data and limited resources for many languages can reduce robustness outside high-resource settings [157], [210]. Real-world deployment also raises governance challenges related to privacy protection, informed consent, record consistency, and local terminology standards [37], [41], [137]. Taken together, these challenges suggest that medical language translation involves more than multilingual language processing. It is also a form of clinical communication where preserving medical meaning is essential.

### 4.4 Medical question answering

Medical question answering is one of the most widely studied applications of Med-LLMs [66]. It plays an important role in supporting knowledge access, clinical communication, and decision support [211]. In this setting, models are expected to respond to natural-language questions about symptoms, diagnoses, treatments, drug use, or biomedical evidence. The task spans two main use contexts. In clinician-facing settings, the goal is to support information retrieval, evidence synthesis, or guideline interpretation during care [212], [213]. In patient-facing settings, models may be used to explain medical information or answer consumer health questions in an accessible language. This distinction matters because the same answer format may not be appropriate across both contexts, and the tolerance for uncertainty, incompleteness, or overstatement differs substantially between professional and consumer use [187], [214]. As a result, medical question answering is not merely a knowledge test for LLMs, but a practical communication task with direct implications for trustworthiness and safety [84], [215].

Current Med-LLMs for medical question answering generally fall into two broad patterns. The first relies on instruction-following models that answer questions directly from internal model knowledge, often performing strongly on benchmark-style exams and short factual questions [212]. Med-PaLM 2 [66] is a representative example and achieved strong performance across multiple tasks in the MultiMedQA [16] benchmark, including multiple-choice medical questions, long-form consumer health questions, and clinical consultation questions [66]. The second pattern emphasizes evidence grounding, often by combining LLMs with retrieval over clinical references, biomedical literature, or curated knowledge sources before answer generation [213]. This design is increasingly important because medical answers are expected to be not only fluent but also aligned with current evidence and able to communicate uncertainty appropriately. Recent work has also moved toward doctor-centered question answering and workflow-aligned conversational settings, where models are evaluated as assistants that support physicians rather than as direct substitutes for clinical judgment [16], [41], [97].

Widely used benchmarks for medical question answering cover several complementary types of clinical knowledge and reasoning tasks [183]. Representative datasets include MedQA [140] and MedMCQA [142], both derived from medical licensing examinations. These benchmarks are widely used to evaluate clinical knowledge and diagnostic reasoning. Other benchmarks focus on understanding biomedical literature and generating evidence-based responses [141]. The MultiMedQA [16] benchmark suite further integrates multiple medical question answering datasets and has been widely adopted to assess the capabilities of LLMs across both professional medical questions and consumer health queries. Evaluation commonly relies on metrics such as accuracy, exact match, and F1 score for structured questions, while text similarity measures, including BLEU [199] or ROUGE [198], are usually used when responses involve longer explanations. However, automatic metrics alone cannot fully capture clinical correctness or safety. Therefore, several studies incorporate expert assessment to evaluate whether generated answers are medically accurate, consistent with available evidence, and appropriate for clinical or patient-facing settings [16], [197], [214].

The main limitations of medical question answering are closely tied to the risks of applying LLMs in high-stakes communication. Models may generate answers that are fluent but incomplete, outdated, or insufficiently grounded in evidence [216]. They may also respond inconsistently when the same question is phrased differently, which is especially problematic in patient-facing settings where users may interpret the response as authoritative advice [217]. In clinician-facing settings, the primary concerns extend beyond readability to include traceability of evidence, appropriate confidence calibration, and consistency with established clinical practice standards [47], [212]. Recent safety studies show that evaluating these systems only for factual accuracy is not enough, because medically unsafe answers can still appear plausible [82], [84], [187]. This makes medical question answering a particularly important application area for Med-LLMs. It highlights the need for better safety-sensitive evaluation, clearer boundaries between assistance and advice, and stronger grounding in clinical evidence and workflow context.

### 4.5 Multimodal medical understanding

Many clinically important questions require information from more than one data modality rather than from a single source alone [67], [218]. In practice, clinicians often interpret medical images together with narrative reports, structured laboratory results, physiological signals, and genomic or other molecular data [219]. This is particularly relevant in radiology, pathology, and precision medicine, where different modalities often contribute complementary information for diagnosis, prognosis, and treatment planning [220], [221]. For Med-LLMs, the value of multimodal modeling lies in its ability to connect these heterogeneous signals and support clinically meaningful interpretation rather than treating each modality as an isolated input [218].

A prominent line of work in this area focuses on integrating medical images with natural language information [112], [222]. Vision-language models link images with associated textual context, such as radiology reports, pathology descriptions, or clinician queries. This design enables systems to support tasks including report generation, image-grounded question answering, and structured interpretation of imaging findings [223]. Early medical adaptations of general vision-language architectures, such as LLaVA-Med [112] and Med-Flamingo [222], demonstrated that domain-specific instruction tuning and medical data curation can substantially improve performance on image-text reasoning tasks. More recent multimodal medical foundation models have extended this paradigm toward broader clinical settings. For example, Med-Gemini [224] integrates diverse medical modalities and has been explored for tasks that combine imaging evidence with clinical narratives and biomedical knowledge. Some emerging studies further suggest that such multimodal frameworks may incorporate molecular or genomic information alongside clinical and imaging data to support applications related to precision medicine [225], [226], [227], [228], [229], [230]. These developments indicate a gradual shift from single-modality image analysis toward more integrated multimodal reasoning in medical AI [36], [231].

The data and evaluation landscape for multimodal medical models remains diverse. Many studies rely on paired image-text datasets, where medical images are linked with radiology reports or clinical descriptions. Widely used resources include datasets derived from large clinical archives, such as Medical Information Mart for Intensive Care Chest X-ray (MIMIC-CXR) [145] or Indiana University Chest X-ray Collection (IU X-Ray) [146], which provide image-report pairs for tasks like report generation and image-grounded question answering. Evaluation typically combines automatic text similarity metrics, such as BLEU [199] and ROUGE [198], with expert assessment, because linguistic overlap alone does not guarantee clinical correctness or appropriate interpretation of imaging findings. Recent benchmarks have also begun to examine whether model outputs are properly grounded in visual evidence, reflecting growing attention to reliability in multimodal medical AI [180], [232], [233]. Even so, no single evaluation framework yet captures all clinically relevant dimensions of multimodal performance.

Genomics represents an important extension of multimodal Med-LLMs beyond conventional image-text settings [227]. Early studies suggest that genomic information can be integrated with clinical narratives, imaging features, and other biomedical data to support tasks such as variant interpretation, disease risk assessment, and patient stratification [162], [225], [227], [228]. This line of research is closely related to radiogenomics, which seeks to connect imaging phenotypes with underlying genomic or molecular patterns [234]. However, incorporating genomic data also introduces additional challenges. Genomic information is highly sensitive, difficult to standardize across institutions, and often probabilistic in interpretation [235]. More broadly, multimodal Med-LLMs must contend with cross-modal misalignment, uneven data quality, and the risk of producing outputs that appear plausible but are not adequately supported by the underlying evidence [180], [232], [233], [235]. These constraints highlight both the promise and the complexity of multimodal medical understanding in clinical applications of Med-LLMs.

### 4.6 Mental health care

Mental health care relies heavily on language-based assessment and ongoing communication, which makes it a natural but high-risk setting for Med-LLM applications [236], [237], [238]. In many situations, clinicians rely on narrative descriptions, conversational exchanges, and patient-reported experiences to understand symptoms and psychological states [239]. For this reason, recent literature generally frames the role of LLMs in mental health as supportive rather than diagnostic. Current systems are most appropriately used to assist with tasks such as summarizing patient-reported concerns or helping communicate mental health information in an accessible language [236]. They may also support low-intensity follow-up communication in certain contexts. These functions can improve access to support and facilitate communication between patients and clinicians [237], [238]. However, they do not replace professional judgment, particularly when situations involve elevated clinical risk or complex therapeutic decisions [168], [236].

Recent studies have explored how Med-LLMs may assist mental health care in practice. One line of work focuses on screening and assessment support [168], [240]. Models analyze interviews, self-reports, questionnaires, or clinical notes to identify linguistic patterns associated with depression, anxiety, or related psychological conditions [239]. An example is MentaLLaMA [165], a mental health-oriented LLM designed for interpretable analysis of mental health-related social media text. Another direction examines conversational support adapted for counseling-style interactions or cognitive behavioral therapy-inspired dialogue [166], [241]. Cognitive Behavioral Therapy-based Large Language Model (CBT-LLM) [166] represents this approach and focuses on cognitive behavioral therapy-based mental health question answering in Chinese. A further area involves psychoeducational and communication support, where models help explain symptoms, summarize counseling content, or assist clinicians with documentation and follow-up communication [236], [238]. Across these forms, the most defensible use case remains assistance to clinicians or service users rather than replacement of therapeutic judgment [237], [238].

Data resources for mental health applications often rely on text-based data derived from interviews, question-answer interactions, and counseling-style conversations [241], [242], [243]. Representative resources include DAIC-WOZ, which contains interview-based data widely used in depression assessment [242]. Other examples include PsyQA [243], which contains question-answer pairs related to mental health knowledge, and dialogue datasets such as CounseLLMe [241] that simulate counseling-style interactions. Evaluation in this area typically combines task-specific metrics with qualitative assessment. Screening-oriented studies often report measures such as accuracy, F1 score, or AUROC, while conversational systems are more frequently examined using language-quality metrics together with expert review [168], [236]. However, recent research emphasizes that automatic metrics alone are insufficient for mental health applications. They cannot fully capture whether a response is safe, supportive, or appropriate for vulnerable users [237], [238]. As a result, many studies now incorporate expert clinical review to assess whether generated responses are clinically appropriate, context-sensitive, and safe for real-world use [236], [237], [244].

Mental health care imposes particularly strict practical constraints on the use of Med-LLMs [238]. Supportive or emotionally responsive language can easily create an impression of understanding that exceeds a model’s actual capacity for clinical judgment. This gap becomes especially concerning in repeated or high-stakes interactions, where users may over-trust the system or rely on it in situations that require professional assessment [244]. Reviews in psychotherapy and psychiatry consistently caution that current LLM-based systems should not be treated as substitutes for licensed care, and recent regulatory discussions reflect similar concerns. Additional challenges arise from the sensitive nature of mental health data and the diversity of language used to describe psychological experiences across cultures and populations [165], [243], [245]. In addition, maintaining consistent safety behavior during extended interactions remains difficult for current systems [82], [187], [246]. For these reasons, mental health care highlights both the potential value of Med-LLMs in supportive communication and the importance of clear boundaries, human oversight, and safety-oriented evaluation in responsible deployment [236], [237], [238].

### 4.7 Traditional Chinese medicine

TCM provides a distinctive application setting for Med-LLMs [169], [247]. Its clinical practice relies on a specialized conceptual system and syndrome differentiation, which link symptoms, signs, constitution, and other contextual information to diagnostic and therapeutic decisions [248]. In this setting, LLM-based applications are not simply Chinese-language versions of general medical question answering [169], [249]. They should also address key elements of TCM reasoning, including pattern differentiation, herbal formula selection, acupuncture-related decisions, and the interpretation of classical medical texts in modern clinical contexts. Recent studies have explored how Med-LLMs can support TCM practice, particularly in consultation settings, knowledge access, and clinical reasoning [110], [169], [187]. At the same time, TCM applications introduce challenges not typically encountered in mainstream biomedical settings. Model outputs must remain consistent with theory-specific terminology and accepted clinical practice [248], [250].

Recent studies have begun to explore several forms of Med-LLMs in the context of TCM [169], [249]. One important direction concerns consultation-oriented question answering, where models are adapted to answer questions from patients or practitioners using TCM knowledge and terminology. Systems such as MedChatZH [249] and HuatuoGPT [110] support multi-turn dialogue grounded in TCM literature and clinical consultation data. In these studies, models are used to support tasks such as syndrome differentiation, acupuncture point selection, and herbal prescription generation. More recent efforts increasingly incorporate structured medical knowledge into the modeling process. Knowledge-enhanced frameworks such as TCM-KLLaMA [172] integrate TCM knowledge graphs with LLMs to improve the consistency and interpretability of generated recommendations. Taken together, these studies suggest that Med-LLMs in TCM are gradually moving beyond general dialogue toward more structured and knowledge-grounded forms of clinical support [249]. An additional emerging direction involves hybrid Med-LLM frameworks that support alignment between TCM practice and Western medicine in integrative care settings [247], [251]. In many real-world clinical environments, patients receive herbal prescriptions together with conventional medications and laboratory-guided treatment [251]. In such scenarios, Med-LLMs may assist clinicians by linking TCM syndrome differentiation with Western diagnoses and medication histories, and by supporting prescription alignment to identify potential inconsistencies or contraindications across treatment systems [172], [248], [252]. Such capabilities may help improve communication between practitioners trained in different medical traditions and facilitate more interpretable integrative decision-making [172], [251].

Evaluation resources for TCM-oriented Med-LLMs are still emerging, with only a small number of dedicated benchmarks currently available [169], [250], [253]. One representative benchmark is TCMBench, which was constructed from TCM Licensing Examination questions and accompanied by a domain-specific scoring method known as TCMScore [250]. Other resources, such as Qibo-Benchmark, have also been proposed to evaluate general knowledge and reasoning in TCM contexts [169]. More recent work has introduced task-oriented benchmarks reflecting clinical scenarios. For example, TCMEval-PA [252] evaluates prescription auditing by assessing the normativity and clinical appropriateness of generated herbal formulas, while TCM-SED [253] focuses on stroke-related TCM tasks, including diagnosis, pattern differentiation, and formula selection. Evaluation commonly relies on metrics such as accuracy, as well as text similarity measures including ROUGE [198] and BERTScore [254] for generative tasks. However, many studies also rely on expert assessment, as automated metrics alone cannot determine whether outputs remain consistent with TCM theory and accepted clinical practice [250], [252].

TCM-oriented applications present several practical constraints for the safe and reliable use of Med-LLMs [251], [255]. Many tasks in this domain do not have a single easily verifiable answer. Syndrome differentiation, prescription selection, and interpretation of classical theory may vary across schools, regions, and clinical styles. In addition, responses that appear plausible may still misrepresent TCM concepts or disrupt the internal logic of herbal formulas [247], [249]. In some cases, they may also produce recommendations that trained practitioners would not consider acceptable [256]. Standardization remains challenging because TCM language draws on diverse sources, ranging from classical literature to modern textbooks and contemporary clinical records. Deployment in broader healthcare environments further introduces requirements related to interoperability, governance, and expert validation [247]. Taken together, these issues show that TCM applications extend Med-LLMs into highly specialized knowledge systems, but their real-world use still depends on domain-grounded evaluation, clinician oversight, and careful governance.

### 4.8 Telemedicine and operational medical support

Telemedicine and operational medical support represent an important application setting for Med-LLMs, particularly in military medicine [257], [258]. In these contexts, medical care is often delivered across distance, under time pressure, and with limited access to specialist support [259], [260]. Military telemedicine has long been used to extend specialist expertise to field hospitals, deployed units, and other austere settings [261]. In these environments, the value of Med-LLMs lies primarily in strengthening clinical communication rather than replacing medical decision-making. They may support remote consultation, structured case summarization, handoff preparation, and the organization of incomplete clinical information [260]. Such functions are closely connected to operational practice, where timely coordination and effective information transfer can help clinicians obtain specialist input and support more informed care decisions [262].

Recent studies have begun to examine how AI systems, including Med-LLMs, may support telemedicine and operational medical care [16], [263]. One important direction involves tele-critical care and remote consultation, where digital platforms connect frontline providers with specialists located away from the point of care [261]. Such systems have been explored in military and other austere environments to extend clinical expertise to field hospitals, deployed units, and emergency response settings. In parallel, recent work has begun to explore AI-assisted decision support. In these settings, Med-LLMs are adapted to interpret clinical guidance and assist with triage decisions, treatment planning, and clinical documentation. Simulation studies suggest that systems aligned with operational medical guidelines, including Tactical Combat Casualty Care (TCCC), may improve decision consistency in selected scenarios [175], [262]. Overall, these developments indicate a gradual shift from communication infrastructure alone toward AI-supported consultation and operational decision support [261], [263].

In contrast to many other Med-LLM applications, evaluation in this area relies less on standardized public benchmarks [263], [264]. One reason is that military and operational telemedicine often involves sensitive data, deployment-specific workflows, and rare but high-consequence scenarios that are difficult to capture in openly shared datasets [257]. As a result, many studies rely instead on simulated case scenarios, implementation studies, and expert-led workflow assessment [175], [261]. Relevant evidence also comes from related resources, including emergency triage tasks, handoff-note studies, and teleconsultation workflows [265], [266]. These alternatives allow researchers to examine whether systems improve communication, support documentation, and assist decision-making in realistic settings. In practice, evaluation therefore depends more on scenario-based testing, usability, and expert review than on benchmark accuracy alone [175], [259].

Telemedicine and operational medical support often take place under demanding conditions [258]. Clinical information is often incomplete, and communication may be delayed or unreliable. In some situations, limited escalation pathways may lead clinicians to over-trust plausible but insufficiently supported recommendations [265]. Model performance may also vary in military and other austere environments because patient populations, available resources, and operational priorities differ from those seen in typical training data [16]. The deployment of Med-LLMs in remote military environments introduces additional constraints. Systems must operate under latency, edge-computing limits, cybersecurity requirements, and strict governance expectations [264], [267]. Telemedicine and operational medical support highlight the potential of Med-LLMs to extend clinical expertise to remote and resource-limited environments, particularly in military settings. Their use in such contexts still requires careful oversight and secure deployment.

## 5 Challenges

Although Med-LLMs have demonstrated broad potential across diverse clinical applications, their reliable deployment in real-world medical settings remains constrained by several persistent challenges. These challenges extend beyond model performance alone and involve issues such as factual reliability, limitations of current evaluation practices, safety and regulatory requirements, vulnerability to distribution shift, and the difficulty of maintaining up-to-date medical knowledge [84]. They also reflect the need for effective human oversight when these systems are integrated into clinical workflows [40]. Taken together, these factors define the central obstacles that must be addressed before Med-LLMs can be deployed at scale in high-stakes medical settings. The following sections, therefore, examine these challenges from six complementary perspectives, spanning reliability, evaluation, governance, robustness, model maintenance, and workflow integration.

### 5.1 Hallucination and factual reliability

Hallucination in LLMs refers to the generation of content that appears plausible but is factually incorrect, unsupported by evidence, or inconsistent with established knowledge [268], [269]. In Med-LLMs, this problem is closely linked to factual reliability, because a response may sound clinically appropriate while still lacking adequate evidentiary support [40]. This issue is particularly important in medical settings, where decisions often depend on accurate interpretation of clinical information [64]. Unlike many general NLP applications, even minor inaccuracies in diagnostic suggestions, treatment recommendations, or medical explanations can have significant consequences. As a result, the reliability of model-generated medical content has become one of the central concerns in the clinical use of Med-LLMs [270].

Hallucination and reduced factual reliability can affect multiple clinical applications [64], [268]. In clinical decision support, models may generate diagnostic reasoning or therapeutic recommendations that are inconsistent with established clinical guidelines and available medical evidence [40], [119]. In higher-risk procedural settings such as surgery or perioperative care, similar hallucinated recommendations may be especially concerning because incorrect suggestions may affect time-sensitive decisions, escalation pathways, or procedure-related planning. In medical question answering and knowledge-access scenarios, hallucinations may appear as incorrect references to biomedical studies or inaccurate descriptions of treatment protocols [271]. In communication-oriented tasks such as mental health support or telemedicine consultation, the problem can be more subtle [272]. Responses may appear reasonable while still providing misleading or overly confident suggestions that are not appropriate for the specific clinical situation. Similar concerns also arise in specialized domains such as TCM [253]. Errors in interpreting theoretical concepts or herbal relationships may produce recommendations that appear plausible but remain inconsistent with accepted clinical practice.

Various strategies have been proposed to mitigate hallucination in Med-LLMs [119], [273]. Approaches such as RAG, integration with structured medical knowledge sources, and alignment-based methods can improve the consistency between generated outputs and available evidence [92], [221], [273]. A further promising direction is to combine RAG with domain-specific knowledge graphs in hybrid grounding frameworks [91], [95]. In such systems, retrieved documents can provide up-to-date external evidence, while knowledge graphs can supply structured relations among diseases, biomarkers, drugs, and treatment pathways [98], [182]. This may be particularly valuable in high-stakes settings such as oncology, where Med-LLMs may need to align generated outputs with tumor staging, biomarker profiles, and regimen-specific contraindications [163], [164], [182]. For example, a hybrid design could help reduce unsupported recommendations by jointly checking retrieved oncology guidelines against graph-structured knowledge of drug interactions and treatment eligibility. However, these strategies do not fully eliminate the problem. Models may still generate unsupported conclusions when evidence is incomplete or retrieved information is misinterpreted, especially in cases involving complex clinical reasoning [100], [274]. These limitations mean that hallucination remains a fundamental challenge to the factual reliability of Med-LLMs. Addressing this issue requires improvements in model design as well as more rigorous evaluation of factual reliability in realistic clinical settings.

### 5.2 Evaluation, benchmarks, and clinical validity

Evaluation remains a central challenge for Med-LLMs because strong benchmark performance does not necessarily translate into reliable clinical use [64], [275], [276]. Much of the current literature still relies on exam-style question sets, reference-based text metrics, or narrowly defined task benchmarks to summarize model capability [277]. These approaches can be useful for measuring specific forms of knowledge recall or output similarity, but they do not adequately capture whether a system is clinically valid, appropriate for a medical task, or safe in practice [131]. Recent commentaries and systematic reviews have argued that Med-LLM evaluation should move beyond leaderboard performance and place greater emphasis on construct validity, task realism, and the clinical meaning of model outputs [278], [279], [280]. This concern is particularly important in medicine, where an answer may appear technically plausible while still being incomplete or clinically inappropriate in specific patient contexts [278], [281].

The limitations of current evaluation become more evident when Med-LLMs are examined across different clinical applications [16]. In the task of medical question answering, high accuracy on licensing-style benchmarks such as the United States Medical Licensing Examination (USMLE) [282] or MedQA [140] can reflect strong recall of medical knowledge, yet these scores may overestimate real-world clinical reasoning ability [275]. Representative benchmark comparisons further show that both frontier general-purpose LLMs and domain-adapted Med-LLMs can achieve strong performance on licensing-style medical evaluations [16], [66]. For example, on the MedQA benchmark of USMLE-style questions, GPT-3.5 achieved 60.2% accuracy, Flan-PaLM reached 67.6%, GPT-4-base reached 86.1%, and Med-PaLM 2 reached 86.5% [66]. However, such benchmark gains should still be interpreted cautiously because exam-style accuracy alone does not establish clinical validity or safe deployment in real-world medical settings [46], [97]. This concern has led several recent studies to question the use of exam-style benchmarks as proxies for clinical competence, noting substantial gaps between benchmark performance and expert judgment [277], [279]. Similar issues arise in other tasks. In multimodal and documentation settings, commonly used text-overlap metrics such as BLEU [199] or ROUGE [198] do not guarantee factual correctness or clinically grounded interpretation [283]. In mental health applications, responses that appear empathetic or well-written do not necessarily ensure that the advice is clinically safe [280], [284]. In telemedicine and operational support settings, evaluation often focuses on how systems perform within realistic clinical workflows rather than on benchmark scores alone [278]. Taken together, these observations suggest that clinical validity is strongly task-dependent and requires evaluation strategies that reflect the specific clinical context.

Recent work is increasingly moving toward more clinically relevant evaluation frameworks [131]. Representative directions include expert review methodologies such as CLEVER, scenario-based assessment frameworks, and human-centered evaluation that focuses on safety, usefulness, and clinical workflow performance [279]. Some studies have also explored LLM-as-a-Judge approaches for clinical summarization, although these methods still depend on carefully designed human reference standards and evaluation rubrics [276], [279], [285]. Despite this progress, a unified framework for evaluating Med-LLMs remains lacking. Existing evidence remains fragmented across different tasks and clinical domains [97], [278], [279]. In addition, many studies still prioritize evaluation convenience over clinical realism [277], [280], [286]. More robust evaluation will require not only stronger benchmarks and metrics, but also broader use of expert-centered and prospectively validated assessment strategies.

### 5.3 Safety, ethics, privacy, and regulation

Beyond technical performance, the use of Med-LLMs also raises important questions about safety, ethics, privacy, and regulatory oversight [287], [288]. A model may appear factually reliable and achieve strong benchmark performance, but these results do not guarantee safe use in high-stakes clinical settings [287], [289]. In medicine, the risks extend beyond incorrect answers alone. They also involve over-reliance on persuasive outputs, unclear responsibility for harmful recommendations, and the handling of highly sensitive patient information [290]. In real clinical deployment, this uncertainty may also create legal liability concerns when harmful outputs influence decisions, particularly if the boundaries between clinician judgment, institutional governance, and system recommendations are not clearly defined. Med-LLMs are increasingly used in settings that involve vulnerable patients and high-stakes clinical decisions. Their use also raises strict requirements for privacy and professional accountability [290], [291], [292]. In patient-facing settings, these requirements extend to informed consent, because patients should understand when LLM-based systems are involved in communication, documentation, or decision support, as well as the limits of those systems in clinical care.

These concerns do not appear in the same way across clinical applications. In mental health settings, safety concerns are closely related to vulnerable users and emotionally sensitive interactions [287]. Model responses may seem supportive but remain clinically inappropriate. In telemedicine and operational medical support, privacy and security can be more difficult to manage, especially in remote or military settings [292], [293]. These environments often involve sensitive health data, limited communication infrastructure, and operationally sensitive information. They also increase the risk of bias and uneven performance [294]. The populations, workflows, and resource conditions encountered in military or austere care settings may differ from those reflected in model training data [151]. Across these settings, accuracy alone is not sufficient for responsible clinical use. Med-LLMs must also protect patients, maintain confidentiality, and reduce the risk of unfair or unsafe outcomes [288], [290], [294].

Recent regulatory developments have made these governance requirements more explicit [295], [296], [297]. The European Commission notes that AI-based software intended for medical purposes may fall within the AI Act’s high-risk framework [298]. In this context, Med-LLMs are expected to meet stricter requirements for safety, transparency, human oversight, and system security [288], [296]. Recent guidance on the AI Act suggests that compliance for high-risk medical AI will require stronger attention to documentation, lifecycle management, and human oversight [295], [296], [298]. In practice, this also points to the need for clearer deployment documentation, such as model cards or related reporting standards, to specify intended use, known limitations, validation scope, and oversight requirements in clinical settings [46], [82], [235]. For Med-LLMs, this points to a broader need for traceability and governance throughout design, validation, and real-world use [287], [291]. At the same time, regulatory expectations remain uneven across jurisdictions and use cases [295], [296], [298].

### 5.4 Robustness, generalization, and distribution shift

Robustness and generalization are difficult to ensure in Med-LLMs, because strong benchmark performance does not necessarily translate into stable behavior in real clinical settings [299], [300]. In practice, training, evaluation, and deployment often take place under different data distributions, creating the risk of distribution shift [301]. This issue is especially important in medicine, where variation in patients, documentation practices, or institutional workflows can change model behavior and affect clinical use. Recent work in medical AI has highlighted distribution shift as a persistent obstacle to robust clinical deployment [301], [302].

This problem becomes clearer when viewed across several representative applications discussed earlier [84]. In medical language translation, model performance may vary across languages, patient expressions, and levels of resource availability [160]. In multimodal tasks, robustness may weaken with changes in image quality, acquisition settings, or accompanying documentation, even if benchmark performance appears strong [303], [304]. Mental health applications make this problem especially visible. Model behavior can shift when the system is used in populations or interaction settings that differ from those seen during training [237], [305]. A similar issue arises in telemedicine and operational medical support, where remote or austere care environments may differ from benchmark settings in patient populations, workflows, and resource conditions [302]. These examples suggest that distribution shift is not limited to any single task. It is a recurring challenge that can undermine both generalization and real-world reliability.

Several strategies may improve robustness, including external validation across institutions, scenario-based testing, and evaluation of model uncertainty [302]. These steps are useful, but they do not eliminate the problem. Clinical data, patient populations, and local workflows continue to change, and many deployment environments are still poorly represented in available training and evaluation resources [302]. Strong benchmark performance does not guarantee stable behavior in real clinical use [300]. The robustness of Med-LLMs must be assessed under changing conditions rather than inferred from results obtained in limited settings. This challenge is also closely related to model updating and maintenance, because changes in knowledge and deployment conditions can directly affect robustness over time.

### 5.5 Knowledge updating, model maintenance, and lifecycle challenges

Medical knowledge is not static, and this creates a distinct challenge for keeping Med-LLMs aligned with current evidence [306]. Unlike the cross-setting mismatch discussed above, the central issue here is change over time. Clinical recommendations may be updated, new safety information may emerge, and institutional practices may gradually shift [306]. As a result, a model that performs well at one point may become less reliable as external knowledge, professional standards, and local workflows evolve [307], [308]. Recent work on real-world clinical AI deployment has emphasized that LLM-based systems cannot be treated as fixed tools once they enter practice [307].

The impact of this problem can be seen in several common medical tasks. In clinical decision support and medical question answering, models may continue to produce answers that no longer match current evidence or updated guidelines [306], [309]. In documentation and summarization tasks, changes in note structure, coding practice, or institutional workflow can make existing model outputs less useful [310]. Similar difficulties can also appear in retrieval-supported systems [119], [273]. Access to external sources may improve timeliness, but reliability still depends on whether the underlying knowledge base is complete, well-maintained, and regularly updated. These examples show that knowledge updating and model maintenance are not peripheral engineering concerns [273], [309], [311]. They directly affect whether Med-LLMs remain clinically reliable after deployment [312].

Several strategies may help reduce these risks, including post-deployment monitoring, version control, and retrieval-based access to updated information [313]. Keeping a medical model aligned with current knowledge requires substantial time, cost, and validation effort [312]. Updating a system may involve new data curation, repeated testing, and additional clinical review before the revised model can be used with confidence [307]. Model updates can also introduce new problems. They may improve performance on the target problem while weakening performance on other tasks [314], [315]. For Med-LLMs, long-term reliability depends on continued access to current knowledge and on the ability to introduce changes in a controlled and clinically accountable manner [311], [312]. Knowledge updating and model maintenance, therefore, remain closely linked to oversight, validation, and workflow integration.

### 5.6 Human oversight, workflow integration, and behavior alignment

Med-LLMs may show strong performance in evaluation settings, but safe clinical use still depends on human oversight and effective workflow design [316], [317]. In medicine, Med-LLMs should be used in settings that allow clinicians to review model outputs, make corrections, and override suggestions when necessary [318]. Their value depends on output quality, compatibility with existing patterns of care, and continued clinician control over their use [319]. Med-LLMs should function as tools within clinical systems rather than as independent decision makers. They must also behave in ways that are appropriate for clinical use, including acknowledging uncertainty, avoiding unwarranted confidence, and supporting escalation when human intervention is needed [320], [321].

These concerns become clearer in common clinical tasks. In clinical decision support, model suggestions may assist reasoning, but interpretation and final judgment must remain with clinicians [318]. In documentation and summarization tasks, the value of model outputs depends on whether they can be effectively reviewed, refined, and incorporated into established clinical documentation practices [322]. Mental health applications make the need for oversight especially clear. Responses may sound supportive while remaining clinically inappropriate, and safe use depends on timely supervision and clear referral mechanisms [272]. In mental health settings, human-in-the-loop oversight may be most appropriate when implemented through tiered review and escalation frameworks [236], [238]. For example, Med-LLMs may be limited to low-risk supportive functions such as psychoeducation, documentation drafting, or summarization, while outputs involving diagnostic interpretation, medication-related suggestions, crisis-related language, or high-confidence therapeutic advice should be routed to qualified clinicians for review before use [238], [244], [272]. Such frameworks may also combine uncertainty signaling, audit trails, and referral-first design to reduce the risk that fluent but unsupported responses are treated as clinically appropriate guidance [235], [279]. Several challenges arise in telemedicine and operational medical support, where remote settings can make supervision and workflow coordination more difficult [261], [318]. In these settings, the same output may be helpful in one context but unsuitable in another [320]. Across these tasks, behavior alignment involves more than accurate output. It also depends on whether the model responds cautiously, defers when appropriate, and remains compatible with the clinical setting.

Several approaches may help address these challenges. Human-in-the-loop review, clear escalation rules, and workflow-aware deployment can improve oversight in practice [316], [323]. However, supervision is not achieved simply by asking clinicians to check model outputs after they are generated. Human review may become superficial if model outputs enter the workflow at the wrong point, prove difficult to judge, or gain acceptance too quickly [322]. For Med-LLMs, behavior alignment needs to be judged in real clinical use rather than assumed from model design alone. A clinically aligned system should support care in a way that clinicians can understand, question, and incorporate into existing workflows [46], [324]. In practical deployment, this reviewability may need to be supported by explanation mechanisms such as evidence traceability, source-linked outputs, or attribution-based analysis of influential inputs. In hybrid Med-LLMs that incorporate structured clinical variables, explainable AI techniques such as SHAP may also help reveal which factors most strongly influence model-supported risk estimates or recommendation components [325]. It should also express uncertainty in a usable way, avoid overconfident recommendations, and support timely referral or escalation in higher-risk situations [133]. In this sense, the effective clinical use of Med-LLMs depends on durable forms of supervision and workflow integration, rather than stronger models alone.

## 6 Future work

Med-LLMs have shown broad promise across a range of medical applications, but future progress will depend on more than further gains in model capability. The next stage of development should place greater emphasis on clinically meaningful evidence and validation, grounded and adaptive Med-LLMs, human-centered clinical integration, and deployment across diverse settings. These priorities reflect a broader shift from isolated performance gains toward systems that can be evaluated, updated, supervised, and used responsibly in clinical practice. Future work should focus on making these systems more reliable, better aligned with clinical needs, and suitable for sustained use in real clinical settings.

### 6.1 Clinical evidence and validation

Future work on Med-LLMs should focus less on extending benchmark coverage and more on building clinically meaningful evidence. Benchmark evaluation and retrospective assessment remain useful, especially for early comparison across models and tasks, but they should not be treated as the main measure of progress [197]. High performance on medical examination questions or isolated task benchmarks does not mean that a model is ready for clinical use [326]. Clinical adoption requires evidence that is closer to real tasks, real users, and real care processes. Recent studies already suggest this shift [279], [319], [326]. Some use blinded physician review to assess complex clinical outputs, while others examine model performance in practical clinical tasks such as documentation and decision support [279].

A more mature validation pathway for Med-LLMs should combine several forms of evidence, rather than rely on any single level of assessment. Task-level evaluation will remain necessary, including benchmark results, scenario-based testing, and expert review, but these should be treated as an entry point rather than the final basis for adoption. They can show whether a model performs well on a defined task, but they are less able to show how that performance will translate into routine clinical use. Future work should also examine how models perform within clinical workflows such as documentation, summarization, triage, referral, and decision support, where value depends on timing, interpretability, and fit with existing care processes [31], [326]. This level of evidence is especially important because many Med-LLM applications are intended to support clinical work rather than to function as isolated prediction tools. A further step is to build stronger prospective and real-world evidence through multi-center validation, post-deployment monitoring, and outcome-aware assessment when feasible [19]. Clinical validation should be understood not simply as improved model testing, but as the generation of evidence needed to support safe, effective, and sustained use in clinical practice.

### 6.2 Grounded and adaptive medical large language models

Future Med-LLMs should be developed as systems that are both grounded in reliable medical evidence and able to adapt to changing clinical knowledge [119]. In medical settings, model outputs should not rely only on internal parametric memory [306]. Medical knowledge changes over time, and clinical practice may also vary across different institutions. A more grounded system can link its responses to retrievable and reviewable sources, which makes clinical checking easier and helps reduce unsupported outputs. At the same time, future systems also need to remain adaptive by staying aligned with updated evidence, institutional protocols, and task requirements over time. Recent reviews of RAG in healthcare have highlighted the same need, showing that Med-LLMs require stronger support from external knowledge sources if they are to remain reliable and usable in practice [92], [273]. Related work on dynamic deployment has likewise shown that medical AI systems, especially LLM-based systems, should not be treated as fixed tools once they enter clinical use [306], [307], [312].

Several future directions follow from this shift. One priority is better integration of external medical knowledge, including clinical guidelines, curated institutional resources, and other high-quality reference sources [92]. Retrieval-augmented approaches are already moving in this direction. Recent medical RAG studies suggest that their value extends beyond factual accuracy to source traceability and long-term maintainability [119], [271]. Another priority is to make Med-LLMs easier to update safely. Model adaptation should support version control, revalidation, and traceable changes as knowledge and practice evolve, rather than relying on static model releases [308]. In practice, the timing of such updates should be guided by changes in clinical guidelines, institutional protocols, and local governance requirements rather than by a fixed technical schedule. The third priority is to extend grounding beyond text-only retrieval. Future systems will likely need stronger task-specific grounding for documentation, decision support, and multimodal clinical reasoning, especially when outputs depend on structured records or image-based evidence. Recent work on iterative medical RAG and hybrid systems that combine fine-tuning with RAG also points in the same direction [123]. Future progress may depend less on any single technique than on system designs that better integrate external knowledge, task adaptation, and ongoing validation. In this sense, the goal is not simply to build stronger Med-LLMs, but to build systems that remain clinically reliable as evidence, workflows, and care environments continue to change.

### 6.3 Human-centered clinical integration

Clinical integration will be a major determinant of whether Med-LLMs become useful in real medical settings. In many medical tasks, the real value of Med-LLMs depends on how its output integrates into clinical workflows, rather than just the capabilities of the model itself [326]. A system may perform well in individual evaluations, but its clinical value remains limited if it reaches the wrong users, enters the workflow at the wrong stage, or creates an additional review burden. Future research should address how Med-LLMs can support clinical roles, integrate into routine workflows, and operate under appropriate human oversight. However, recent studies suggest that this type of clinical integration remains limited in practice [316], [319], [327]. For example, in tasks such as clinical documentation or decision support, the usefulness of Med-LLMs often depends on how well they fit existing workflows rather than on model performance alone [40].

The more useful direction for future development is to design Med-LLMs around specific forms of clinical collaboration rather than around generic model capability. Systems designed for different clinical tasks often require different outputs, interface structures, and levels of autonomy [328]. In clinical use, clinicians need to recognize uncertainty, revise model suggestions when necessary, and retain control over final decisions. This also means that workflow design should be treated as a central part of model development rather than as a later implementation step [329]. Med-LLMs are more likely to be adopted when they support review, revision, handoff, and traceability within existing clinical processes, instead of adding a separate layer of work. In this sense, future progress will depend on developing forms of human-AI collaboration that can operate safely and responsibly in clinical practice.

### 6.4 Deployment across diverse clinical settings

Deployment of Med-LLMs will require more than evidence from a single institution or a small set of standardized settings [330]. Real clinical environments differ in patient populations, languages, workflows, infrastructure, and organizational capacity. As a result, a system that appears useful in one setting may not transfer smoothly to another. Therefore, future deployment of Med-LLM systems should account for heterogeneity rather than assuming that clinical environments are broadly uniform. Recent implementation reviews show that real-world integration of LLMs in clinical workflows remains limited and uneven, while work on global health equity has highlighted how current development and deployment remain concentrated in high-income settings [316], [326], [331]. This observation also highlights an important issue for future research. Effective deployment depends on model quality as well as careful evaluation and adaptation across different clinical settings.

Several issues should be addressed to support deployment across diverse clinical settings. Broader external validation will be essential before large-scale adoption, particularly across different institutions and patient populations [93]. Deployment planning should also incorporate fairness and equity from the outset rather than treating them as secondary considerations. Future systems should be assessed for uneven performance across demographic groups, languages, and care environments, especially in settings that are underrepresented in current development pipelines [332]. Deployment infrastructure should account for privacy, security, and governance requirements [291]. In practice, implementation often depends as much on organizational capacity as on technical performance. In remote military or other austere settings, future deployment may also require lightweight Med-LLMs that can be integrated with edge computing infrastructure to support real-time applications under constrained connectivity and hardware conditions. Such designs may be particularly important for field hospitals, deployed units, and other operational environments where low latency, local processing, and rapid clinical response are necessary. In such environments, continual adaptation may need to rely less on frequent full-model retraining and more on lower-cost strategies such as retrieval-based knowledge updating, parameter-efficient adaptation, and centralized revalidation, so that edge-side systems can remain lightweight while still benefiting from controlled model maintenance. In addition, Med-LLMs intended for real-world use should remain maintainable after deployment. Grounded systems need continued monitoring and updating if they are to remain reliable as evidence, workflows, and local practice continue to change. The long-term goal is to support context-aware deployment that remains reliable and appropriate across diverse clinical settings, rather than assuming that systems can be applied uniformly.

## 7 Conclusions

Med-LLMs have shown broad potential across a range of clinical tasks, including decision support, documentation, and patient communication. At the same time, their clinical value cannot be determined by model capability alone. Current evidence suggests that reliable use of Med-LLMs in practice depends on more than performance in controlled settings. It relies on the strength of supporting evidence, alignment with current medical knowledge, and effective integration into clinical workflows. By bringing together recent work on applications, challenges, and emerging directions, this review highlights the key conditions for meaningful use of Med-LLMs in clinical practice.

The development of Med-LLMs is increasingly centered on clinical validity, system design, and effective use in practice, rather than on model performance itself. Future advances will rely on stronger clinical evidence, better alignment with evolving knowledge, and more effective integration into routine care. It will also require approaches to deployment that account for differences across institutions, patient populations, and resource conditions. The focus should be on supporting systems that remain reliable in clinical practice while adapting over time and being used under appropriate oversight. Med-LLMs will have clinical impact only if they can function as part of a broader clinical system that remains accountable, maintainable, and responsive to real clinical needs.

## Abbreviations

AI: Artificial intelligence

EHR: Electronic health record

LLMs: Large language models

Med-LLMs: Medical large language models

MoE: Mixture of experts

NLP: Natural language processing

PEFT: Parameter-efficient fine-tuning

RAG: Retrieval-augmented generation

SFT: Supervised fine-tuning

SSMs: State space models

TCM: Traditional Chinese medicine

## Ethics approval and consent to participate

Not applicable.

## Authors’ contributions

YS, PC, TT, and KFL conceived and designed the topic of this review. YS and LY drafted the manuscript. YS, LY, ZL, XZ, AK, LSW, and YY performed the literature search and review. YS prepared the figures and tables. PC, TT, KFL, YL, TZ, SSG, ZW, RH, KL, and SKI reviewed and revised the manuscript. All authors contributed to the interpretation and final preparation of the manuscript. All authors read and approved the final manuscript.

## Funding

This work was supported by the Macao Polytechnic University (RP/FCA-14/2023), the Science and Technology Development Funds (FDCT) of Macao (0033/2023/RIB2), the Joint Research Funding Program between FDCT and the Department of Science and Technology of Guangdong Province (FDCT-GDST) (0009/2024/AGJ), the Joint Research Fund between FDCT and the Ministry of Science and Technology of China (FDCT-MOST) (0106/2025/AMJ) and the Anusandhan National Research Foundation (ANRF) under the Partnerships for Accelerated Innovation and Research (PAIR) programme under the sanction order (ANRF/PAIR/2025/000029/PAIR).

## Competing interests

The authors declare that they have no competing interests.

## Data Availability

Not applicable.
